# Chlamydial genes shed light on the evolution of photoautotrophic eukaryotes

**DOI:** 10.1186/1471-2148-8-203

**Published:** 2008-07-15

**Authors:** Burkhard Becker, Kerstin Hoef-Emden, Michael Melkonian

**Affiliations:** 1 Botanisches Institut, Universität zu Köln, Gyrhofstr. 15, 50931 Köln, Germany

## Abstract

**Background:**

Chlamydiae are obligate intracellular bacteria of protists, invertebrates and vertebrates, but have not been found to date in photosynthetic eukaryotes (algae and embryophytes). Genes of putative chlamydial origin, however, are present in significant numbers in sequenced genomes of photosynthetic eukaryotes. It has been suggested that such genes were acquired by an ancient horizontal gene transfer from Chlamydiae to the ancestor of photosynthetic eukaryotes. To further test this hypothesis, an extensive search for proteins of chlamydial origin was performed using several recently sequenced algal genomes and EST databases, and the proteins subjected to phylogenetic analyses.

**Results:**

A total of 39 proteins of chlamydial origin were retrieved from the photosynthetic eukaryotes analyzed and their identity verified through phylogenetic analyses. The distribution of the chlamydial proteins among four groups of photosynthetic eukaryotes (Viridiplantae, Rhodoplantae, Glaucoplantae, Bacillariophyta) was complex suggesting multiple acquisitions and losses. Evidence is presented that all except one of the chlamydial genes originated from an ancient endosymbiosis of a chlamydial bacterium into the ancestor of the Plantae before their divergence into Viridiplantae, Rhodoplantae and Glaucoplantae, i.e. more than 1.1 BYA. The chlamydial proteins subsequently spread through secondary plastid endosymbioses to other eukaryotes. Of 20 chlamydial proteins recovered from the genomes of two Bacillariophyta, 10 were of rhodoplant, and 10 of viridiplant origin suggesting that they were acquired by two different secondary endosymbioses. Phylogenetic analyses of concatenated sequences demonstrated that the viridiplant secondary endosymbiosis likely occurred before the divergence of Chlorophyta and Streptophyta.

**Conclusion:**

We identified 39 proteins of chlamydial origin in photosynthetic eukaryotes signaling an ancient invasion of the ancestor of the Plantae by a chlamydial bacterium accompanied by horizontal gene transfer. Subsequently, chlamydial proteins spread through secondary endosymbioses to other eukaryotes. We conclude that intracellular chlamydiae likely persisted throughout the early history of the Plantae donating genes to their hosts that replaced their cyanobacterial/plastid homologs thus shaping early algal/plant evolution before they eventually vanished.

## Background

Transfer of genes between different genomes is now recognized as widespread and has been postulated to play an important role during evolution [1,2]. Gene transfer between different species is generally termed horizontal gene transfer (HGT) or lateral gene transfer (LGT). HGT is especially common in prokaryotes [1], however, it has become recently clear it also occurs frequently in phagotrophic protists [2]. In addition, in eukaryotes a large number of genes were transferred from the evolving plastid and mitochondrion to the nuclear genome. This type of gene transfer has been named intracellular gene transfer (IGT, [3]) or endocytotic gene transfer (EGT, [4]). HGT or EGT leads to an exchange of genes between distantly related organisms creating genetic chimeras. It allows the recipient to acquire new characters de novo and therefore challenges the traditional evolutionary concept that evolution proceeds by modification of existing genetic information. As is evident most clearly in EGT no gene appears to be immune to transfer, however, most of the recently transferred genes (HGT) appear not to include housekeeping genes [5,6]. HGT is generally detected by phylogenetic analyses (reviewed in [7]) and is generally considered as a form of noise, obscuring phylogenetic signal and therefore interfering with the reconstruction of the evolution or a group of organisms [5,8]. However, Huang and Gogarten [3] suggested that ancient horizontal gene transfers are helpful for elucidating the evolutionary history of a group of organisms.

Chlamydiae are well known donors for horizontal gene transfer events [9]. Chlamydiae are an ancient group of obligate intracellular bacteria [10], probably most closely related to the Verrucomicrobia [11]. The presence of *Chlamydia*-type genes in plants (or plant-like genes in Chlamydiae) has been reported repeatedly [12-16]. Three different explanations have been forwarded: 1) HGT of plant genes to a chlamydial ancestor [12], 2) HGT of chlamydial genes to plants [15] and 3) an ancient phylogenetic relationship between Chlamydiae, Cyanobacteria, and chloroplasts [13]. A list of chlamydial proteins with high similarity to plant proteins by Brinkman et al. [13] included 37 proteins involved in diverse functions such as protein and fatty acid synthesis, glycolysis, and nucleotide metabolism. Whereas a list by Horn et al. [14] using proteins of the *Parachlamydia*-related symbiont of acanthamoebae (recently proposed as Candidatus *Protochlamydia amoebophila *[17]) as query in BLAST searches found 137 proteins from *Protochlamydia *with high similarity to plant proteins. Recently, a list of 14 chlamydial genes present in Viridiplantae and *Cyanidioschyzon merolae *(Rhodoplantae) has been published by Huang and Gogarten [16]. These authors postulated an ancient EGT event to explain the large number of genes transferred [16]. Several additional genomes (including those of two diatoms, three chlorophytes and several additional cyanobacteria), and a large number of additional ESTs (including those of two glaucophytes and several red and green algae) have been recently sequenced. Therefore, we have reinvestigated the phylogenetic relationships between plastids, Cyanobacteria and Chlamydiae using an extended taxon sampling. We show that 39 chlamydial type genes are present in Glaucoplantae, Rhodoplantae, Viridiplantae and Bacillariophyta indicating an ancient origin for these chlamydial genes. However, the distribution of chlamydial proteins among different photoautotrophic eukaryotes is complex requiring an in depth phylogenetic analysis.

## Results

### A significant number of chlamydial genes occur in eukaryotic algae

**Figure 1:**
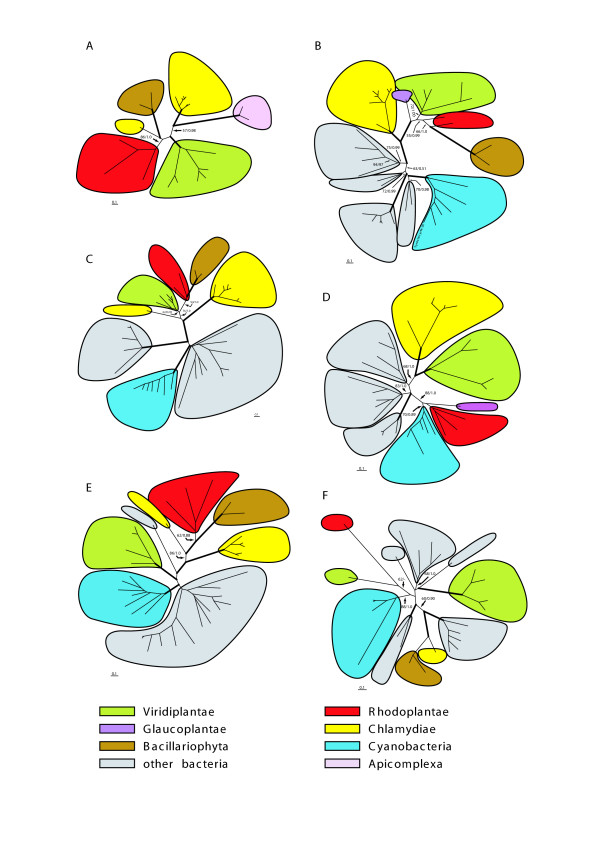
**Phylogenetic analysis of chlamydial genes in photoautotrophic eukaryotes**. Unrooted maximum likelihood trees of single-gene data sets. Evolutionary models of all data sets: WAG+I+Γ, except for Fig. 1E: RtREV+I+Γ ([85]. Support values: maximum likelihood bootstrap/posterior probability; branches in bold: ML bootstrap > 95% and posterior probability of 1.0. Scale bars = substitutions per site. For enlarged trees with taxon names, see Additional File 4. (A) Glycerol-3-phosphate acyltransferase (EC 2.3.1.15; 21 taxa, 281 amino acid positions). (B) tRNA delta(2)-isopentenylpyrophosphate transferase (EC 2.5.2.8; 38 taxa, 240 positions). (C) 2-C-methyl-D-erythritol 4-phosphate cytidyltransferase (*isp*D; EC 2.7.7.60; 38 taxa, 198 positions). Rhodoplantae, Bacillariophyta and Viridiplantae group with the Chlamydiae. (D) Putative ribosome release/recycling factor (COG0233; 30 taxa, 171 positions). (E) Ribosomal large subunit pseudouridine synthase (EC 4.2.1.70; 38 taxa, 262 positions). (F) Folylpolyglutamate synthase (EC 6.3.2.17; 26 taxa, 208 positions).

To search for genes of putative chlamydial origin in eukaryotic algae, the genomes of two diatoms (*Thalassiosira pseudonana*, *Phaeodactylum tricornutum*), three green algae (*Ostreococcus tauri*, *Ostreococcus lucimarinus, Chlamydomonas reinhardtii*), and the red alga *Cyanidioschyzon merolae *were screened for the presence of protein-coding genes most similar to genes in the genome of Candidatus *Protochlamydia amoebophila *(see Methods). The initial screen identified 89 putative chlamydial proteins in these algae (Additional File 1). Maximum likelihood phylogenetic analyses were performed with each of the 89 proteins using a taxon sampling that included other eukaryotic algae and chlamydiae, embryophytes, cyanobacteria, and other bacteria (an average of 30 taxa for each protein); 37 proteins revealed a monophyletic lineage (with 70–100% bootstrap support (BS) for 33 proteins, and 57–68% BS for 4 proteins) consisting of chlamydiae and at least one of the three plastid-containing eukaryotic lineages represented by genomes (diatoms, green algae/embryophytes, and red algae; Table 1) to the exclusion of all other proteins. For two additional proteins (putative glycerol-3-phosphate acyltransferase (Fig. 1A), hypothetical protein pc0324 (no 32, Table 1)), the phylogenetic trees contained only chlamydiae and plastid-containing eukaryotes because proteins with significant similarities to the chlamydial proteins could not be retrieved from any other taxa. Among the remaining 50 phylogenetic trees, several trees also displayed a topological sister group relation between chlamydiae and plastid-containing eukaryotes, however, without bootstrap support (not shown).

**Table 1:** Proteins of putative chlamydial origin in plastid-containing eukaryotes.

	Gene^1)^	Bootstrap support	Comments
	**(Chlamydiae, Plantae)**		
1	**Asparaginyl-tRNA synthetase** gi|46445980	74	
2	Aspartate aminotransferase gi|46446319	98	**(CB F) **100% ML
3	ATP/ADP translocase	n. a.	Only in intracellular parasites and plastid-containing eukaryotes
4	**tRNA delta(2)-isopentenylpyro-phosphate transferase** gi|46446877	99	
5	**Diphosphate-fructose-6-phosphate 1-phosphotransferase** gi|46446514	58	
	**(Chlamydiae, Viridiplantae, Rhodoplantae)**		
6	Isopentenyl monophosphate kinase (ISPE) gi|46447223	74	
7	**Queuine tRNA-ribosyltransferase** gi|46446428	100	*Dictyostelium *sister to **[P]**
8	**Putative 7-dehydrocholesterol reductase** gi|46446854	94	Only eukaryotes except *Protochlamydia *and *Coxiella, Coxiella *sister to *Protochlamydia *(87% ML)
9	Putative 23S rRNA (Uracil-5-)-methyltransferase gi|46447632	100	
10	Putative 4-diphosphocytidyl-2C-methyl-D-erythritol synthase (ISPD) gi|46445961	99	**(CB F) **73% ML
11	**Hypothetical protein pc1328** gi|46446962	82	
12	Putative glycerol-3-phosphate acyltransferase gi|46446952	n. a.	Only present in Chlamydiae and plastid containing eukaryotes
13	Probable polyribonucleotide nucleotidyltransferase gi|46446277	100	**(CB F) **100% ML
14	**Probable S-adenosyl-methyltransferase** gi|46445945	62	
15	**Putative tRNA pseudouridylate synthase I** gi|46445962	79	
16	3-oxoacyl-(acyl carrier protein) synthase (FABB) gi|46446872	100	Bacillariophyceae in eubacterial clade
17	**Putative endopeptidase (ATP-dependent serine protease) La** gi|46446096	83	
18	Probable tyrosine-tRNA ligase gi|46446803	97	Bacillariophyceae in bacterial clade
19	Probable isoamylase gi|46446740	99	
20	**Probable S-adenosyl-methyltransferase** gi|46445945	81	Bacillariophyceae in eukaryotic clade
21	Putative oligoendopeptidase F gi|46446812	73	
	**(Chlamydiae, Viridiplantae)**		
22	**Phosphate transporter** **gi|46445733**	88	
23	Phosphoglycerate mutase gi|46399436	94	
24	Probable gcpE protein (ISPG) gi|46446374	91	**(CB G R) **100% ML
25	Enoyl-(acyl carrier protein) reductase (FABI) gi|46446786	100	**(CB R)**
26	**DNA mismatch repair protein (MUTS)** gi|46446855	54	**(CB R) **95% ML
27	**Putative lipoate-protein ligase** gi|46447472	98	*Dictyostelium *sister to **[B V]**
28	Gut Q protein gi|46447416	100	
29	Malate dehydrogenase gi|46447406	92	NADP^+^-dependent plastidial homologue of the Viridiplantae
30	**Putative ribosome recycling factor** gi|46447510	68	(**CB R G**) 88% ML
31	**Putative tyrosine/tryptophan transport protein** gi|46445802	99	
32	**Hypothetical protein pc0324** gi|46445958	n. a.	Only present in Viridiplantae and Protochlamydia
33	**Hypothetical protein pc0378** gi|46446012|	99	
34	Probable 3-deoxy-manno-octulosonate cytidylyltransferase (CMP-KDO synthetase) gi|46400100	86	
	**(Chlamydiae, Rhodoplantae)**		
35	**Probable 23S RNA-specific pseudouridine synthase D** gi|46445989	96	
36	**Hypothetical protein pc0339** gi|46445973	100	*Geobacter *sister to **[V]**
37	**Cysteinyl-tRNA synthetase** gi|46446869	100	*Leptosira *sister to **[C P]**
	**(Chlamydiae, Bacillariophyceae)**		
38	**Putative folylpolyglutamate synthase** gi|46447260	99	
39	Transketolase gi|46447148	70	*Dictyostelium *sister to **[B], [V G CB]**

39 proteins were identified by ML analyses. The proteins are grouped according to the observed tree topologies in the maximum likelihood analyses. The bootstrap support (ML) for the indicated clades is given. For 20 genes (in bold letters) this is to our knowledge the first detailed phylogenetic analysis. See Additional File 2 for an extended version of Table 1 including tree topologies.

^1) ^The accession number and annotation for the gene from *Protochlamydia *is given. n. a. not applicable, A Apicomplexa, B Bacillariophyta, C Chlamydiae except Protochlamydia, CB Cyanobacteria, CP Chlorophyta, F Firmicutes, G Glaucoplantae, P Protochlamydia, R Rhodoplantae, SP Streptophyta, V Viridiplantae.

The proteins of putative chlamydial origin in plastid-containing eukaryotes perform a broad spectrum of functions with isoprenoid, fatty acid and carbohydrate metabolism, and phosphate homeostasis featuring prominently (Additional File 1). All are encoded on the host's nuclear genome, and most (but not all, e.g. CMP-KDO synthetase) are predicted to be targeted to the plastid. Metabolic pathways involving putative chlamydial proteins reveal a chimerical origin (some genes of the plastidic isoprenoid and fatty acid biosynthesis pathways are of cyanobacterial origin).

### Complex distribution of chlamydial genes among plastid-containing eukaryotes

**Figure 2:**
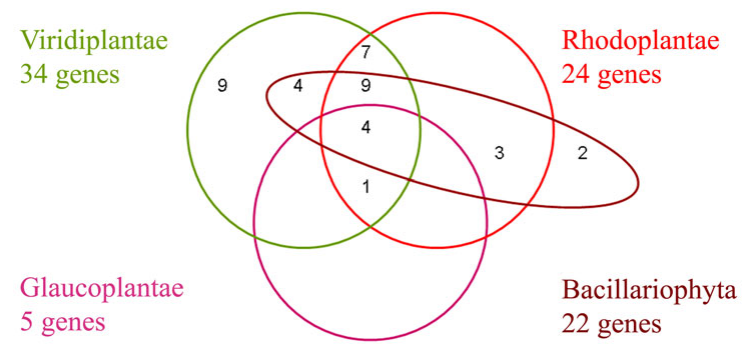
Venn diagram showing the number of chlamydial proteins shared by different photoautotrophic eukaryotes.

The distribution of the 39 chlamydial proteins among four groups of photosynthetic eukaryotes (Viridiplantae (green algae and embryophytes), Rhodoplantae (red algae), Glaucoplantae (glaucophytes), and Bacillariophyta (diatoms)) is shown in a Venn diagram (Fig. 2). The largest number of chlamydial proteins (34) was found in the Viridiplantae, followed by the Rhodoplantae (24), Bacillariophyta (22) and Glaucoplantae (5). It should be noted that the low number of chlamydial proteins recovered from the Glaucoplantae presumably relates to that fact that no genome of this small, yet phylogenetically important, algal lineage has been sequenced to date and the chlamydial proteins had to be retrieved from the limited EST (cDNA) data available for two Glaucoplantae, *Cyanophora paradoxa *and *Glaucocystis nostochinearum*. The Viridiplantae contain the largest number of unique chlamydial proteins (9), followed by the Bacillariophyta (2), whereas no unique chlamydial proteins were recovered from the Rhodoplantae and Glaucoplantae. Viridiplantae and Rhodoplantae share 21, Viridiplantae and Bacillariophyta 17, and Rhodoplantae and Bacillariophyta 16 chlamydial proteins. Both Viridiplantae and Rhodoplantae share the same 5 chlamydial proteins with the Glaucoplantae (four of which are also present in the Bacillariophyta). Seven chlamydial proteins are unique to Viridiplantae and Rhodoplantae, 4 to Viridiplantae and Bacillariophyta, and 3 to Rhodoplantae and Bacillariophyta. Chlamydial proteins involved in the same biosynthetic pathway may be differentially distributed in the different algal lineages. In the plastidic isoprenoid pathway, the chlamydial genes *isp*D and *isp*E (nos 10 and 6 respectively, Table 1) are present in the Rhodoplantae and Viridiplantae, whereas the chlamydial *isp*G (gcpE; no 24, Table 1) is only present in the Viridiplantae and Bacillariophyta; the Rhodoplantae and Glaucoplantae contain the cyanobacterial homologue of *isp*G. Similarly, in the fatty acid synthesis pathway, the chlamydial gene *fab*B (no 16, Table 1) is present in both Rhodoplantae and Viridiplantae, whereas the chlamydial *fab*I (no 25, Table 1) is restricted to the Viridiplantae (and Bacillariophyta and Apicomplexa); the Rhodoplantae contain the cyanobacterial *fab*I. The complex distribution pattern of chlamydial proteins in the different lineages of photosynthetic eukaryotes called for an in depth analysis of the phylogeny of each chlamydial protein using a broader taxonomic sampling that included diverse chlamydiae, cyanobacteria and other groups of bacteria such as proteobacteria and Firmicutes (representative trees in Fig. 1A–F, summary in Table 1 and Additional File 2).

### Chlamydial genes in photosynthetic eukaryotes signal an ancient, horizontal gene transfer event

The presence of a relatively small number of genes from another domain of life in a genome is usually regarded as signalling horizontal gene transfer (HGT). If a large number of foreign genes that have a single origin are involved, an endosymbiotic gene transfer (EGT) is often the favoured hypothesis. EGT is a special case of HGT, in which the donor (endosymbiont) transfers its genome or part of it to the acceptor (host) and physically persists for an extended period of time within the host. If the donor disappears from its host, it may be difficult, if not impossible, to distinguish between HGT and EGT. As HGT involves two genomes, the direction of gene flow is not *a priori *known, e.g. the presence of chlamydial proteins in photosynthetic eukaryotes could either signal HGT from chlamydiae to eukaryotes or HGT from eukaryotes to chlamydiae. Phylogenetic analyses can often resolve this conundrum. However, in photosynthetic eukaryotes an additional level of complexity is added by the presence of nuclear-encoded cyanobacterial genes. Since the 39 chlamydial proteins are, with a few notable exceptions (three chlamydial genes in *Dictyostelium *that may have originated by another HGT; nos 7, 27,39; Table 1), confined to plastid-containing eukaryotes, it is also conceivable that the presence of proteins of putative chlamydial origin in these eukaryotes simply reflects their phylogenetic relationship to the respective cyanobacterial homologues rather than an HGT between chlamydiae and eukaryotes, the former perhaps obscured by loss or modification of the respective genes in extant free-living cyanobacteria. Again phylogenetic analyses can help to reach an informed decision. The presence of cyanobacterial genes in the host genome has another connotation; it may indicate that HGT perhaps did not take place between the chlamydial and eukaryotic genomes directly but between chlamydial and cyanobacterial genomes either before or after the cyanobacterial endosymbiosis (note that the latter scenario requires the simultaneous presence of cyanobacteria and chlamydiae within the host). In this case the chlamydial genes were transferred to the nuclear genome by EGT from the genome of the cyanobacterial endosymbiont.

For 9 of the 39 chlamydial proteins detected in plastid-containing eukaryotes, a cyanobacterial homologue could not be recovered (Fig. 1A and Table 1). Analyses of the remaining 30 chlamydial proteins revealed no specific phylogenetic relationships of the cyanobacterial and chlamydial proteins to the exclusion of other bacterial homologues (e.g. Fig. 1B–F). In fact, the cyanobacterial proteins sometimes formed strongly supported clades with proteins of other bacteria to the exclusion of proteins from chlamydiae (and their associated homologues in plastid-containing eukaryotes). Often, but not always, the cyanobacterial proteins were recovered as sisters to the respective proteins of the Firmicutes (e.g. polyribonucleotide nucleotidyltransferase (bootstrap support: 100/1.0 ML/PP; no 13, Table 1) and aspartate aminotransferase (100%/1.0 ML/PP; no 2, Table 1)). The cyanobacteria/Firmicutes sister group relationship had previously been inferred from multigene phylogenies (e.g. Battistuzzi et al. [18] using 32 proteins (7,597 positions) from 54 different bacterial strains)). Although this result does not rule out an ancient relationship between cyanobacteria and chlamydiae, it strongly suggests that the chlamydial proteins present in photosynthetic eukaryotes are not simply modified cyanobacterial proteins and thus are not of cyanobacterial but of chlamydial origin. That cyanobacterial homologues of chlamydial proteins can be readily identified as such when present in photosynthetic eukaryotes, is exemplified by three proteins with differential distribution in photosynthetic eukaryotes, the cyanobacterial homologue being associated with Glaucoplantae and/or Rhodoplantae (nos 24, 26, 30; Table 1, Fig. 1D), while the chlamydial homologue is present in Bacillariophyta and/or Viridiplantae.

Phylogenetic analyses of the 39 chlamydial proteins also provide insight about the direction of HGT between chlamydiae and plastid-containing eukaryotes. Although two chlamydial proteins occur only in chlamydiae and plastid-containing eukaryotes (see above), and another chlamydial protein (ATP/ADP translocase, no. 3, Table 1) is present only in intracellular bacterial parasites and plastid-containing eukaryotes, all other chlamydial proteins have homologues among several major groups of bacteria and are thus embedded in the bacterial radiation. Conversely, the chlamydial proteins identified here are confined to plastid-containing eukaryotes among the eukaryote radiation (with the exception of the three chlamydial proteins found in *Dictyostelium*, see above). The presence of chlamydial genes in eukaryotes with secondary plastids (Bacillariophyta and Apicomplexa (Table 1)), and chlorarachniophytes (results not shown)) suggests that these genes have spread from Plantae (comprising Glaucoplantae, Rhodoplantae, and Viridiplantae) to other eukaryote supergroups such as heterokonts, alveolates, and Rhizaria together with their secondary, eukaryotic endosymbionts. In conclusion, chlamydial proteins in plastid-containing eukaryotes are most likely derived from chlamydiae by HGT/EGT.

When and how often did HGT/EGT between chlamydiae and plastid-containing eukaryotes occur? For chlamydial proteins with homologues in all three lineages of the Plantae (5 chlamydial proteins; Table 1), a sister group relationship was recovered between chlamydiae and Plantae for each protein (e.g. Fig. 1B). This suggests that HGT from chlamydiae to plastid-containing eukaryotes predated the divergence of the Glaucoplantae, Rhodoplantae and Viridiplantae.

For 8 additional proteins, a sister group relationship between chlamydiae and Rhodoplantae+Viridiplantae was observed (with BS support ranging from 79–100%; e.g. Fig. 1C; nos 6, 7, 10, 11, 15–18, Table 1) strongly suggesting that HGT for these proteins predated the split between Rhodoplantae and Viridiplantae. It is anticipated that most, if not all of these 8 proteins will also contain homologues in the Glaucoplantae, which are, however, currently unknown reflecting the paucity of genome information in the Glaucoplantae (see above). For another 7 chlamydial proteins in which a monophyletic origin of chlamydiae, Rhodoplantae, and Viridiplantae was recovered (Table 1), either the branching order among chlamydiae, Rhodoplantae and Viridiplantae was unresolved (BS support ≤ 63%; nos 9, 13, 14, 19–21; Table 1) or the (single) protein occurred only in chlamydiae and plastid-containing eukaryotes (putative glycerol-3-phosphate acyltransferase; Fig. 1A). With the addition of more taxa, in particular from the Glaucoplantae, the branching order between chlamydiae and plastid-containing eukaryotes for these 7 chlamydial proteins may be resolved and a monophyletic origin of the chlamydial proteins in plastid-containing eukaryotes likely be revealed. Only one chlamydial protein (putative 7-dehydrocholesterol reductase: no 8, Table 1) deviates from this scheme (for this protein, long branch attraction between Rhodoplantae and chlorophytes, and the absence of any bacterial proteins other than Candidatus *Protochlamydia amoebophila *and *Coxiella *may have obscured phylogenetic relationships among taxa; Table 1).

For chlamydial proteins that display a sister group relationship between chlamydiae and Viridiplantae (13 proteins; Table 1), either the homologue from Rhodoplantae is missing (7 proteins; nos 23, 27–29, 31–33) or the Rhodoplantae homologue is specifically associated (often as a sister group) with cyanobacteria (e.g. Fig. 1D; 6 proteins; nos 22, 24–26, 30, 34; Table 1). The first situation may reflect selective loss of chlamydial genes in the rhodoplant lineage, the second offers several contrasting explanations (see below).

Chlamydial proteins that reveal a sister group relationship between chlamydiae and Rhodoplantae (3 proteins; Table 1) also display homologues in the Viridiplantae, whose positions, however, remained unresolved in the phylogenetic trees (Fig. 1E; nos 35–37; Table 1).

Finally, chlamydial proteins showing a sister group relationship between chlamydiae and Bacillariophyta to the exclusion of Plantae (putative folylpolyglutamate synthase (Fig. 1F) and transketolase; nos 38 and 39 respectively; Table 1) either contain the three lineages of Plantae in a monophylum together with cyanobacteria (transketolase) or two lineages of Plantae (Rhodoplantae and Viridiplantae) together with cyanobacteria, Firmicutes and other bacteria in an unresolved radiation (Fig. 1F). It is likely that at least the transketolase of the Bacillariophyta originated by an HGT involving a chlamydial donor different from the donor(s) that contributed the chlamydial genes in the Plantae (see also [19]).

The overall conclusion from the phylogenetic analyses is that the vast majority of the 39 chlamydial proteins detected in photosynthetic eukaryotes during this study (a minimum of 31, perhaps all, except one) presumably entered the Plantae by HGT from chlamydiae before the divergence of the Glaucoplantae, Rhodoplantae and Viridiplantae.

Another important question relates to the possible nature of the donor in the HGT/EGT of the chlamydial genes into plastid-containing eukaryotes. For 7 chlamydial proteins, Candidatus *Protochlamydia amoebophila *was the only member of the chlamydiae in the trees; proteins from other chlamydiae could not be retrieved from the data bases (Fig. 1F; Table 1). The phylogenetic tree for one of these proteins, asparaginyl-tRNA synthetase (no 1; Table 1), also included all three lineages of the Plantae, and in this case, *P. amoebophila *formed a weakly supported sister group to the Plantae + Bacillariophyta (58% BS support for Plantae + Bacillariophyta; Table 1). For the other 6 proteins, *P. amoebophila *was either in an unresolved position among the photosynthetic eukaryotes (3 proteins; nos 8, 9, 20; Table 1), was the only bacterial protein in a tree, that, in addition to *P. amoebophila*, comprised only one lineage of Plantae (Viridiplantae; hypothetical protein pc0324; no 32; Table 1) or was a sister group to the Bacillariophyta (e.g. Fig. 1F; 2 proteins; nos 36, 38; Table 1).

Four of the 5 trees that contained all three lineages of Plantae, displayed, in addition to *P. amoebophila*, other chlamydiae (Fig. 1B; nos 2–5; Table 1). In one of the 4 trees (aspartate aminotransferase; no 2; Table 1), the chlamydiae were paraphyletic (with *P. amoebophila *as a sister group to the Plantae (BS 77% for Plantae), in two others (nos 4 and 5; Table 1) they were monophyletic, with *P. amoebophila *as their first divergence, and the chlamydiae in sister group position to the Plantae. In one tree (ATP/ADP translocase; no 3; Table 1) relationships among the chlamydiae were unresolved. In the 4 trees, the chlamydiae (except *P. amoebophila*) exhibited relatively long branches, and in the case of the aspartate aminotransferase, this may have caused artificial attraction of these chlamydiae to a very long branch comprising all other bacteria (except cyanobacteria) rendering the chlamydiae paraphyletic (not shown).

Extending the comparison to the other 28 phylogenetic trees that contained chlamydial proteins from both *P. amoebophila *and other chlamydial taxa, again two topologies emerged: a monophyletic chlamydiae (12 proteins; Table 1) in which *P. amoebophila *always represented the first divergence within the chlamydiae (e.g. Additional File 3D), or a paraphyletic (sometimes polyphyletic) divergence of the chlamydiae (16 proteins; Table 1) with *P. amoebophila *either in a sister group position (with or without BS support) to the chlamydial proteins of the plastid-containing eukaryotes (e.g. Additional File 3E; 9 proteins; nos 6, 7, 18, 22–24, 27, 31, 35), or positioned (albeit without resolution) within the Plantae (3 proteins; nos 12, 15, 16, Table 1). For 4 chlamydial proteins a sister group topology between the chlamydiae (excluding *P. amoebophila*) and either a single clade of plastid-containing eukaryotes (twice with Viridiplantae (nos 21, 28; Table 1), and once with Rhodoplantae (no. 19; Table 1); in nos 19 and 21 without bootstrap support; Table 1) or with two clades of plastid-containing eukaryotes (Viridiplantae and Bacillariophyta; no 26; Table 1) was observed. In all 10 phylogenetic trees in which the chlamydiae were paraphyletic and *P. amoebophila *was sister to the plastid-containing eukaryotes (see above), the chlamydiae (excluding *P. amoebophila*) exhibited much longer branches than the branches of both *P. amoebophila *and the plastid-containing eukaryotes (which may have drawn them closer to the long branches of other bacteria or conversely resulted in short branch attraction of *P. amoebophila *and the plastid-containing eukaryotes), again likely rendering the chlamydiae paraphyletic (see above). Support for the monophyly of the chlamydiae is strongest (≥ 95% BS; 6 proteins; nos 13, 17, 30, 33, 37, 39) in cases in which the branch lengths of *P. amoebophila *and the other 4–8 chlamydiae are of comparable lengths (not shown) or in which branch lengths of the plastid-containing eukaryotes and the bacteria (other than chlamydiae) are of similar lengths (RNA delta(2)-isopentenylpyrophosphate transferase; Additional File 3B; no 4; Table 1). When support for the monophyly of the chlamydiae was strong (≥ 95% BS), so was support for the sister group relationship between chlamydiae and plastid-containing eukaryotes (BS ≥ 96%; 5 proteins; nos 4, 13, 17, 33, 37; Table 1).

From these data it is concluded (1) that the chlamydiae are monophyletic, (2) that the apparent paraphyly (polyphyly) of chlamydiae seen in some trees is the result of a long branch (or short branch) attraction artifact because of largely differing evolutionary rates between the genes of *P. amoebophila *and the later diverging intracellular chlamydial parasites of animal hosts, (3) that the later diverging chlamydiae lost several genes that *P. amoebophila *(and the plastid-containing eukaryotes) retained, (4) that the donor of the chlamydial genes found today in the plastid-containing eukaryotes was related to the common ancestor of *P. amoebophila *and other chlamydiae, and thus (5) that the HGT/EGT of chlamydial genes into plastid-containing eukaryotes occurred more than 700 MYA, the estimated time of divergence of *P*.*amoebophila *from other chlamydiae [14]. The apparent ancient origin of the chlamydial genes in plastid-containing eukaryotes is independently supported by the recovered monophyly of five chlamydial proteins found to date in all three lineages of Plantae (with 58 – 100% BS support for the monophyly of the Plantae; Table 1). The divergence time between Rhodoplantae and Viridiplantae has been variously estimated, based on molecular clock analyses, to be at least 1,100 MYA [20-22].

### Phylogeny of chlamydial proteins supports the monophyly of the Plantae, but can chlamydial proteins also shed light on subsequent algal evolution?

Because chlamydial genes apparently entered the nuclear genome of plastid-containing eukaryotes before the divergence of the three major lineages of Plantae (see above), their fate can be traced through algal evolution and subsequent secondary endosymbioses in much the same way as that of plastidial proteins of cyanobacterial origin. Whereas the monophyly of the Plantae is rarely questioned today (but see [23] and reviews by [24,25]), the sequence of evolutionary divergence of the three principal types of plastids that correspond to the three lineages of Plantae, namely cyanelles (Glaucoplantae), rhodoplasts (Rhodoplantae) and chloroplasts (Viridiplantae) still remains unresolved. All possible topologies of the three lineages of plastids (or the corresponding Plantae) have been recovered in phylogenetic analyses, i.e. a sister group of Rhodoplantae and Viridiplantae (R+V; [26-28]), a sister group of Glaucoplantae and Rhodoplantae (G+R; [29,30]), or a sister group of Glaucoplantae and Viridiplantae (G+V; [26,31-33]. Often plastid and host trees yielded conflicting results and statistical support was low, taxon sampling was insufficient, especially in analyses of host genes (10–16 taxa of Plantae; [33]) or apparently long-branch attraction artefacts prevailed.

Chlamydial proteins do not (yet) shed light on the sequence of divergence of the three lineages of Plantae, likely because too few such proteins are currently known from the Glaucoplantae. For the 5 chlamydial proteins present in all three lineages of the Plantae, none of the three topologies obtained for the Plantae was significantly supported by bootstrap values (Table 1). Two proteins supported a G+R sister group (nos 3 and 5, Table 1), whereas two other proteins supported a R+V sister group (nos 2 and 4, Table 1). The fifth chlamydial protein (Asparaginyl-tRNA synthetase, no. 1, Table 1) supported a G+V sister group, however the Viridiplantae were paraphyletic in this analysis (Table 1). For two additional proteins, chlamydial homologues were present only in the Viridiplantae, whereas the Glaucoplantae and Rhodoplantae contained the cyanobacterial homologues (nos 24 and 30, Table 1). The distribution of the latter two proteins among the three lineages of Plantae is most parsimoniously explained by either a G+R or R+V topology. To further evaluate the different topologies, phylogenetic analyses with concatenated data sets using *P. amoebophila *and two other chlamydiae as outgroup were performed. In a 4-protein analysis (aspartate aminotransferase, ATP/ADP translocase, tRNA delta(2)-isopentenylpyrophosphate transferase and diphosphate-fructose-6-phosphate 1-phosphotransferase; nos 2–5, Table 1) using 576 aa positions, a moderate (71%) BS support for a sister group relationship between Rhodoplantae and Viridiplantae (R+V) was obtained (data not shown). When more protein sequences of chlamydial origin will become available from the Glaucoplantae, chlamydial proteins may provide a unique opportunity to address the pattern of evolutionary divergence in the three lineages of Plantae.

The complex distribution pattern of chlamydial proteins in the plastid-containing eukaryotes in combination with the detailed phylogenetic analyses (see above), suggest that chlamydial proteins were differentially lost from individual lineages of Plantae. Of the 13 chlamydial proteins recovered from Viridiplantae only, in seven cases chlamydial homologues were absent from Rhodoplantae suggesting that these were lost in the Rhodoplantae after their divergence from the Viridiplantae. For two of these proteins (nos 23 and 28, Table 1), the chlamydial homologues were also absent and thus presumably lost in the chlorophyte sublineage of the Viridiplantae, perhaps indicating that these proteins perform specific functions confined to the streptophyte sublineage of the Viridiplantae. It should be noted that taxon sampling in the Rhodoplantae was mostly limited to *Cyanidioschyzon merolae *and *Galdieria sulphuraria*, two members of a specialized group of thermophilic red algae with extremely small genomes (i.e. the Cyanidiophyta). It is thus possible, although not perhaps likely, that the absence of these chlamydial proteins may be confined to this sublineage of the Rhodoplantae and that more "typical" Rhodoplantae will be shown to contain them. For the other 6 chlamydial proteins confined to the Viridiplantae, the Rhodoplantae contain a cyanobacterial homologue (nos 22, 24–26, 30, 34, Table 1). This can be interpreted in different ways. It may be argued that this is indicative of a separate origin of chlamydial proteins in the Viridiplantae (but see results and discussion above). Alternatively, it indicates that both the cyanobacterial and the chlamydial homologues were present in the ancestor of the Plantae and persisted until after the divergence of Rhodoplantae and Viridiplantae, when they were differentially lost (the cyanobacterial homologue in the Viridiplantae and the chlamydial homologue in the Rhodoplantae (and likely also in the Glaucoplantae; nos 24 and 30, Table 1)). In case of the phosphate transporter (no. 22, Table 1), it is likely that the cyanobacterial homologue was lost only from the streptophyte sublineage of the Viridiplantae as the chlorophyte sublineage contains a cyanobacterial homologue (not shown). It is important to stress that in no case cyanobacterial and chlamydial homologues of the same protein have been found in a single taxon of Plantae (Table 1) suggesting that their functions (within the plastid) are redundant and their occurrence thus mutually exclusive. Although several different scenarios may explain the simultaneous presence of cyanobacterial and chlamydial homologues of a larger number of proteins throughout the early history of the Plantae, the most intriquing is certainly the simultaneous presence of two different intracellular bacteria throughout the early history of the Plantae, i.e. cyanobacteria and chlamydiae (see Discussion).

### Origin of chlamydial proteins in the Bacillariophyta: a hidden secondary endosymbiosis

For 22 chlamydial proteins putative homologues were identified in the two sequenced genomes of the Bacillariophyta (Fig. 2). Two of these chlamydial proteins apparently occur exclusively in the Bacillariophyta and not in the Plantae (nos 38 and 39, Table 1) which instead contain cyanobacterial homologues of these proteins (e.g. Additional File 3F). Since it is well established that the Bacillariophyta (and the heterokonts in general) obtained their plastids through a secondary endosymbiosis involving a rhodoplant symbiont (reviews by [34-36]), either the rhodoplant symbiont of the heterokonts still contained both homologues (which were later differentially lost in the Rhodoplantae and the heterokonts) or, at least in the case of transketolase (no. 39, Table 1) more likely, the chlamydial protein was independently acquired by HGT in the Bacillariophyta. The latter scenario is favored for transketolase because *Dictyostelium *is a sister to the two Bacillariophyta, and all three lineages of Plantae lack the chlamydial homologue.

For the remaining 20 chlamydial proteins, the Bacillariophyta formed a sister group with either the Rhodoplantae (10 proteins; RB clade, Table 1) or the Viridiplantae (10 proteins, VB clade; Table 1). Both topologies received similar support values, however, BS values were sometimes low. The four chlamydial proteins that were detected in all lineages of the Plantae, revealed exclusively an RB clade with variable BS support (94, 66, 80 and 55% BS values; Table 1). Of the 16 chlamydial proteins that occurred in both Rhodoplantae and Viridiplantae, 9 were also present in the Bacillariophyta (Fig. 2). Of those 9 proteins, one protein (no. 10; Table 1) revealed a strongly supported RB clade (94% BS), four proteins a VB clade (nos 6–8, 13, supported by BS values of 68, 54, 100, and 86%, respectively; Table 1), and the remaining 4 proteins exhibited either one or the other topology, but without BS support (nos 9, 11, 12, 17; Table 1). Of the 13 chlamydial proteins that were found only in the Viridiplantae, four were also detected in the Bacillariophyta (Fig. 2), for two of which the Bacillariophyta formed a clade with the Viridiplantae (no. 24; 92% BS; Table 1) or a sister group with the streptophyte sublineage of the Viridiplantae (no. 26; 100% BS; Table 1), for one protein, the VB topology was unsupported (no. 27; Table 1) and for the remaining protein, the Bacillariophyta were sister to the Apicomplexa (no. 25; 64% BS; Table 1). All three chlamydial proteins that were detected only in the Rhodoplantae (Fig. 2), also displayed homologues in the Bacillariophyta: for two proteins, a well-supported RB clade was recovered (nos 35 and 37; 97 and 96% BS support, respectively; Table 1), for the third protein, the Rhodoplantae and Bacillariophyta formed a clade that also included *P. amoebophila *with 100% BS support (no. 36; Table 1). It should be noted that in two of the chlamydial proteins revealing a well-supported RB clade (nos 3 and 35), the RB clade contained, in addition to the two Cyanidiophyta, also members of the Rhodophyta (the second major lineage of the Rhodoplantae according to [37]).

**Figure 3:**
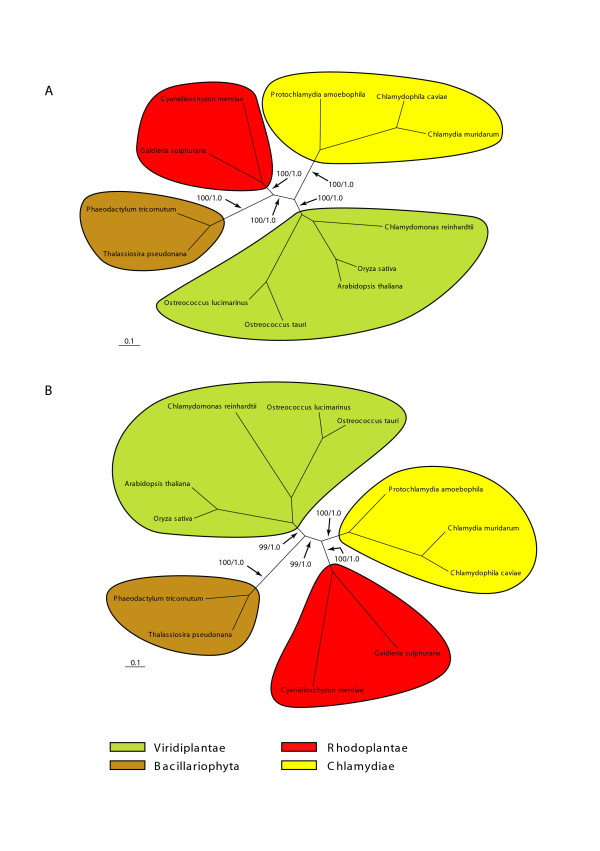
**Phylogenetic analyses of chlamydial proteins in the Bacillariophyta support the occurrence of two independent HGT/EGT events**. Unrooted maximum likelihood trees of concatenated data sets. Support values: maximum likelihood bootstrap/posterior probability. Scale bars = substitutions per site. (**A**) Concatenated data set of seven proteins showing relationship of diatoms to rhodoplants (12 taxa; 2675 amino acid positions). (**B**) Concatenated data set of five proteins showing a relationship of viridiplant and diatom genes (same 12 taxa as in Fig. 3A; 1736 amino acid positions). For a complete list of genes used in the two concatenated analyses see Additional File 5.

To study the phylogeny of the chlamydial proteins from the Bacillariophyta in more detail, unrooted maximum likelihood phylogenetic analyses of concatenated data sets were performed (Fig. 3). In the first analysis (Fig. 3A), 7 chlamydial proteins that individually had revealed an RB topology (with or without BS support; nos 1–4, 10, 12, 17; Table 1) were concatenated and subjected to phylogenetic analysis using 12 taxa (2675 amino acid positions; Fig. 3A). The taxa were selected to maximize taxon congruence with the second data set of proteins displaying a VB topology (Fig. 3B), i.e. two Bacillariophyta, the chlorophyte and streptophyte sublineages of the Viridiplantae (5 sequences), the Rhodoplantae (2 sequences, except for no. 10 for which only one rhodoplant sequence was recovered) and, if possible, 3 sequences of chlamydiae were included. The RB clade was recovered with 95% BS values (ML) and 1.0 posterior probability in the Bayesian analysis (Fig 3A). In a second analysis (Fig. 3B), 5 chlamydial proteins that had individually revealed a VB topology (nos 6, 7, 9, 11, 13; Table 1; it should be noted that only protein no. 13 received BS support for a VB clade) were concatenated and subjected to phylogenetic analyses using the same 12 taxa as in the concatenated analysis of the proteins with an RB topology (1736 amino acid positions; Fig 3B). In this analysis, the VB topology was strongly supported (>95% BS values in ML and 1.0 posterior probability in the Bayesian analysis; Fig. 3B). Although in the individual analyses of the 5 chlamydial proteins, the position of the Bacillariophyta with respect to the sublineages of the Viridiplantae was not resolved and BS values for 4 of the 5 proteins were below 50% (Table 1), the concatenated data set clearly indicated that the 5 chlamydial proteins of the Bacillariophyta were sister to the respective proteins of the Viridiplantae, i.e. they diverged before the split of the Viridiplantae into its chlorophyte and streptophyte sublineages (Fig. 3B).

From these data the following tentative conclusions are drawn: (1) The Bacillariophyta obtained chlamydial genes from two sources of photosynthetic eukaryotes, the Rhodoplantae and the Viridiplantae, (2) it is very likely that the chlamydial proteins forming RB clades were obtained during the secondary endosymbiosis of a rhodoplant symbiont into a heterokont (or chromalveolate) host, (3) there is preliminary evidence that the endosymbiosis of the rhodoplant occurred before the Rhodoplantae diverged into Cyanidiophyta and Rhodophyta (although this needs to be further studied using a larger taxon sampling in the Rhodoplantae), (4) chlamydial proteins of the Bacillariophyta forming VB clades were obtained by HGT or EGT from a viridiplant alga before the Viridiplantae diverged into Streptophyta and Chlorophyta, (5) since the number of chlamydial proteins forming VB clades in the Bacillariophyta found so far is roughly equivalent to the number of chlamydial proteins forming RB clades, it is suspected that EGT rather than HGT was the source of the viridiplant genes, the endosymbiont (or its plastid) being no longer present in extant heterokonts (or chromalveolates), (6) it cannot currently be decided which of the two secondary endosymbioses preceded the other. If the above conclusions are valid, the two secondary endosymbioses may have taken place during the same time period. Two chlamydial proteins involved in essential plastidic pathways (fatty acid biosynthesis, FABI; isoprenoid biosynthesis, ISPG; nos 24 and 25, Table 1) occur in the Bacillariophyta and Viridiplantae, but not in the Rhodoplantae (both Cyanidiophyta and Rhodophyta; tree not shown), which harbor the cyanobacterial homologues (Table 1). For ISPG, two Glaucoplantae also contain the cyanobacterial homologue. If the rhodoplant symbiont of the Bacillariophyta also contained the cyanobacterial homologues (the most parsimonious scenario), the latter must have been replaced in the Bacillariophyta by the chlamydial homologue from the viridiplant symbiont, requiring the simultaneous presence of both eukaryotic symbionts in the host.

## Discussion

Chlamydiae are a group of obligate intracellular bacteria that are well-known pathogens of animals and humans and have been studied for decades [38,39]. More recently, a large diversity of previously unrecognized chlamydiae was discovered in the environment (e.g. [40-42]), where they have been found in intracellular associations with diverse eukaryotic hosts ranging from amoebae to invertebrates. Phylogenetic and phylogenomic analyses of chlamydiae indicated that "environmental chlamydiae" represent a sister group of present-day chlamydiae pathogenic in animals, that separated from their common ancestor more than 700 million years ago [14], suggesting that the ancestor already lived intracellularly in eukaryotes. Recently, the Verrucomicrobia, which have been estimated to comprise up to 10% of the soil bacterial flora and have also been found in aquatic systems including lakes, marine sediments and hot springs but also live associated with eukaryotes, were identified as the closest known free-living relatives of chlamydiae [11,43].

While chlamydiae are obligate intracellular pathogens/symbionts in many eukaryotes, they have not been discovered to date in Plantae or secondary plastid-containing eukaryotes. However, when the first chlamydial genomes were sequenced, a surprisingly high proportion of genes with highest similarity to plant genes were discovered [12]. This finding, in conjunction with the obligate intracellular lifestyle of chlamydiae, sparked a number of studies that aimed to elucidate the phylogenetic history of the plant-like chlamydial genes. The conclusions ranged from proposals that the chlamydial proteins in plants simply reflected an ancient phylogenetic relationship between chlamydiae and cyanobacteria (and thus plastids) [13,44] to an HGT between chlamydiae and plants with either the chlamydiae [15,45,46] or plants [47,48] proposed as donors.

While the present work was in progress, Huang and Gogarten [16] published data, based on phylogenomic analyses of the rhodoplant *Cyanidioschyzon merolae *to identify chlamydial homologues, which suggested that at least 21 genes were transferred between chlamydiae and the ancestor of Rhodoplantae and Viridiplantae. They concluded that the donor was most similar to present-day *Protochlamydia *and that the relatively high number of genes transferred suggested an ancient chlamydial endosymbiosis with the ancestral primary photosynthetic eukaryote. These authors also hypothesized that the chlamydial endosymbiont was perhaps necessary to facilitate the establishment of the cyanobacterial endosymbiont, explaining the apparent uniqueness of primary plastid evolution and providing independent evidence for the monophyly of eukaryotes harboring primary plastids. The results and conclusions by [16] are corroborated by the present analyses (for differences in interpretation of phylogenetic analyses of some putative chlamydial proteins between the two studies, see Additional File 4). Using an extended phylogenomic analyses that included three chlorophyte (*Chlamydomonas reinhardtii*, *Ostreococcus tauri *and *O. lucimarinus*), a second rhodoplant (*Galdieria sulphuraria*), and two diatom genomes (*Thalassiosira pseudonana *and *Phaeodactylum tricornutum*), and sequences retrieved from EST databases (in particular from the two glaucoplants, *Cyanophora paradoxa *and *Glaucocystis nostochinearum*), as well as model-specified phylogenetic analyses of all proteins recovered, the number of chlamydial proteins now identified in photosynthetic eukaryotes has doubled [39 vs 19 (21) in Huang and Gogarten's analyses (two of their proteins are believed to be false positives; see Additional File 4)], which is still likely an underestimate. This is corroborated by a recent study that provided a list of 55 genes in Plantae of putative chlamydial origin [49], including 24 of the 39 proteins of likely chlamydial origin described in this study. Different data mining strategies including different e-value cut offs in the initial BLAST analyses, different phylogenetic methods used and the overall more conservative approach taken in this study may account for the observed different numbers of genes reported.

Most significantly, five chlamydial proteins (instead of previously only one, i.e. ATP/ADP translocase) are now represented in the three lineages of Plantae and their phylogenetic analyses all revealed monophyly of the Plantae as well as their sister group relationship to chlamydiae. Although *P. amoebophila *was often recovered as a sister to the Plantae, in such topologies other chlamydiae were either absent (suggesting loss of the respective genes in such chlamydiae) or the chlamydiae were paraphyletic, the latter likely an artifact of long-branch attraction (see Results). It is concluded that the donor of presumably all (except one) of the chlamydial genes found today in plastid-containing eukaryotes was related to the common ancestor of *P. amoebophila *and other chlamydiae, and thus that the HGT/EGT of chlamydial genes into plastid-containing eukaryotes was an ancient event that occurred more than 700 MYA, the estimated time since divergence of *P*.*amoebophila *from other chlamydiae [14]. Evidence has also been presented (see Results) that the acceptor of this HGT/EGT event was related to the ancestor of the Plantae, i.e. that HGT/EGT of chlamydial genes occurred before the divergence of the Glaucoplantae, Rhodoplantae and Viridiplantae.

**Figure 4:**
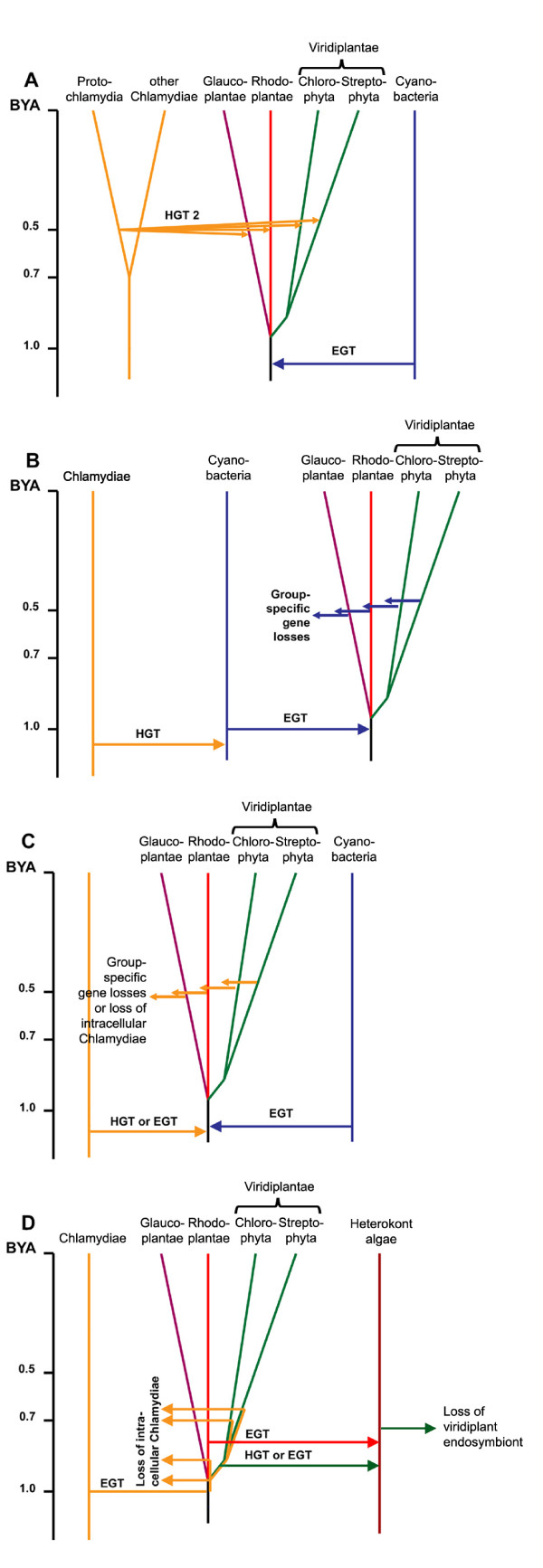
**Scenarios to explain the simultaneous presence of cyanobacterial and chlamydial protein homologues in photosynthetic eukaryotes**. (A) Multiple HGTs of chlamydial genes from a single donor into different photosynthetic eukaryotes. (B) Single or multiple HGTs of chlamydial genes into the cyanobacterial ancestor of plastids and group-specific gene losses from different photosynthetic eukaryotes. (C) HGT or EGT from intracellular chlamydiae to the cyanobacterial endosymbiont of a photosynthetic eukaryote and group-specific chlamydial gene losses from different photosynthetic eukaryotes. In a variation of this scenario the intracellular chlamydiae donate genes by EGT or HGT to the eukaryotic host little before or at the time of cyanobacterial endosymbiosis and group-specific multiple gene losses of chlamydial genes. (D) Origin of chlamydial proteins in heterokont algae. Two secondary endosymbioses are shown involving sequentially a viridiplant and a rhodoplant symbiont. The endosymbiosis of a cyanobacterium has been omitted from Figure 4D for clarity.

The simultaneous presence of cyanobacterial and chlamydial homologues of a larger number of proteins throughout the early history of the Plantae can, in principle, be explained by four alternative scenarios (Fig. 4A–C): (1) the first scenario (multiple HGTs from a *Protochlamydia*-type donor into different photosynthetic eukaryotes; Fig. 4A) can be rejected (see above), because it is in conflict with the presence of chlamydial genes of the same origin in the common ancestor of the Plantae (see above). (2) The second scenario (Fig. 4B) assumes that chlamydial genes were transferred from a chlamydial donor(s) by a massive single or by multiple HGTs into the cyanobacterial ancestor of plastids before primary endosymbiosis, followed by multiple losses of chlamydial genes in the different lineages of photosynthetic eukaryotes. This scenario fails to explain the complete absence of chlamydial genes in any extant cyanobacterium (unless this gene transfer was a unique event, see below) and cannot provide a rationale for retention of two functional homologues (of cyanobacterial and chlamydial origin) of a protein over extended periods of time in the cyanobacterial symbiont (or plastid). Although HGT among prokaryotes is rampant [eg. [50-53]] it is difficult to envision what kind of adaptive advantage would be gained in a free-living cyanobacterium upon acquisition of, e.g., the ATP/ADP translocase [54]. (3 – 4) In the third and fourth scenario (Fig 4C), HGT or EGT of chlamydial genes occurred during the same time period as EGT of the cyanobacterium into a eukaryotic host. Either there was massive bulk transfer of genes from an intracellular chlamydial bacterium to the host or cyanobacterial endosymbiont with subsequent differential loss of these genes in the different lineages of Plantae (HGT) or the chlamydial bacterium persisted as an intracellular symbiont/parasite over extended but varying periods of time in the three lineages of Plantae (EGT) and different chlamydial proteins were successively recruited by the eukaryotic hosts. The latter scenario is favored because the simultaneous presence of chlamydial and cyanobacterial homologues of enzymes with the same function over an extended period of time presumably requires compartmentalization, i. e. residence in their original environment, the respective symbionts. Whether gene transfer occurred first from the intracellular chlamydial bacterium to the host nucleus with later retargeting of the protein to the cyanobacterium (plastid), as hypothesized by [16] or directly between the two types of intracellular bacteria (chlamydial bacterium and cyanobacterium), with gene transfer from the plastid to the host nucleus occurring later (favored here) or perhaps using both routes, remains unknown and possibly depended on the exact timing of the two endosymbioses and the intracellular location of the symbionts relative to each other.

A co-existence of a chlamydial bacterium and the evolving plastid in the eukaryotic host over an extended time period may seem unlikely; however, multiple intracellular bacteria occur in extant protists, and recently, Heinz et al. [55] provided evidence for the beneficial association of two intracellular bacteria (a chlamydial bacterium and a proteobacterium) in a free living *Acanthamoeba*. There is also evidence for mobile DNA in intracellular bacteria of eukaryotic hosts [56,57] and the recent discoveries of conjugation machineries in intracellular rickettsiae and chlamydiae have been particularly enlightening [58,59]. Based on phylogenetic and bioinformatic analyses, Ogata et al. [59] concluded that genes involved in conjugative DNA transfer have been exchanged by HGT between the ancestors of rickettsiae and environmental chlamydiae in a eukaryotic host, likely an amoeba. This may have been the same HGT event that resulted in the transfer of ATP/ADP translocase (in total five paralogous *tlc *genes), the hallmark enzyme of the intracellular ATP-parasitism of rickettsiae and chlamydiae, from the ancestor of chlamydiae to the ancestor of rickettsiae [45,46,60,61]. Although it has recently been suggested that the plastid paralogue (NTT1) of the ATP/ADP translocase might have been derived from the mitochondrial ancestor (and thus from a rickettsia-type α-proteobacterium; [62]), this is unlikely because almost all of the 39 chlamydial proteins identified to date in plastid-containing eukaryotes presumably originated in an intracellular chlamydial symbiont/parasite residing in the Plantae (this study), while the origin of mitochondria occurred much earlier (none of the 39 chlamydial proteins, with three exceptions [*Dictyostelium*], has been found in other eukaryotes).

This study also offered the first opportunity to trace the spread of chlamydial proteins through secondary plastid endosymbioses using phylogenomic information from the two diatoms *Thalassiosira pseudonana *and *Phaeodactylum tricornutum*. Phylogenetic analyses of the 20 chlamydial proteins recovered from the diatom proteomes led to the conclusion that chlamydial proteins originated by two separate secondary endosymbiotic events, one involving a viridiplant and a second involving a rhodoplant symbiont, only the latter surviving in extant photosynthetic heterokonts. It is also concluded that both secondary endosymbioses were ancient, presumably occurring before the major radiations in both lineages of Plantae took place (see Results and Fig. 4D).

Analysis of the genome of *Thalassiosira pseudonana *gave the first indication that the diatom proteome contained a surprisingly large number of proteins that matched only to Viridiplantae (i.e. *Arabidopsis thaliana*; 865 proteins), more than four times as many as to the rhodoplant *Cyanidioschyzon merolae *[63]. Even when one considers that the *A. thaliana *proteome is more than four times larger than the *C. merolae *proteome, there would still be about equal numbers of diatom proteins matching either to Viridiplantae or Rhodoplantae. The numerical ratio of diatom proteins with similarity to either Viridiplantae or Rhodoplantae was even more biased towards Viridiplantae, when animals (*Mus musculus*) were replaced by the cyanobacterium *Nostoc *sp. PCC 7120 (2023 "green" proteins vs. 254 "red" proteins) in proteome comparisons, perhaps suggesting that the contribution of viridiplant proteins to the diatom proteome extends well beyond the plastid proteome. These results were corroborated in a comparative proteome approach using more than 5,000 non-redundant EST sequences from the pennate diatom *P. tricornutum *[64]. The genomes of the early diverging heterokonts *Phytophthora sojae *and *P. ramosum *displayed a large number of genes (855) supporting a photosynthetic ancestry of these presumably aplastidial protists, and again a significant portion of these genes revealed best matches to Viridiplantae [65].

Although the origin of the heterokont plastid from a red alga has been established beyond doubt using phylogenetic and phylogenomic approaches [66-71], phylogenomic analyses of the "green" proteins in heterokonts/chromalveolates have only recently been initiated [65,71-74]. Li et al. [71] used 5,081 expressed sequence tags of the haptophyte *Emiliania huxleyi *and a phylogenomic approach including genome and EST data from other algae, animals, plants and bacteria to identify the source of endosymbiotic gene transfer for 19 non-paralogous proteins using maximum-likelihood phylogenetic analyses: 17 genes were of the expected red algal origin, whereas for two genes (chlorophyll a synthase, phosphoribulokinase [PRK]) Viridiplantae were the sister group of the heterokonts/chromalveolates to the exclusion of the Rhodoplantae. Similar results regarding the phylogeny of PRK were obtained by Petersen et al. [72]. Whereas Petersen et al. [72] suggested that PRK in heterokonts/chromalveolates originated by a single non-endosymbiotic HGT from a green alga to a rhodoplast-containing protist, Li et al. [71], referring to the earlier controversial discussion about a putative green algal ancestry of the apicoplast-encoded elongation factor *tufA *[75] and the apicomplexan mitochondrial-targeted *cox2a *and *cox2b *subunits [76], raised the possibility that "green" proteins in heterokonts/chromalveolates may have originated by EGT from a green alga that was endosymbiotic in a heterokont/chromalveolate. From their data, Li et al. [71] concluded that the red algal contribution, however, was at least an order of magnitude larger than that of green algae. In a related study, Nosenko et al. [73] identified 30 different plastid-targeted proteins from two EST-libraries of the tertiary plastid-containing dinoflagellate *Karenia brevis*. Of 22 proteins whose evolutionary origins could be resolved, 13 were of rhodoplant, while 6 were of viridiplant origin. These authors suggested that a major influx of viridiplant genes occurred early in the evolution of heterokonts/chromalveolates, and since all "foreign" genes acquired by chromalveolates before their divergence into heterokonts/alveolates were derived from a single donor (a viridiplant), one possible explanation would be the presence of a green algal endosymbiont in the chromalveolate ancestor prior to the rhodoplant endosymbiosis [71]. The phylogenetic trees derived to date from most of the "green" proteins in chromalveolates (e.g. PRK, periplasmic serin protease IV, soluble inorganic pyrophosphatase, γ-Tocopherol O-methyltransferase) suggest that EGT occurred before the divergence of Chlorophyta and Streptophyta, in accordance with the results presented in this study. In conclusion, heterokonts/chromalveolates seem to have obtained chlamydial proteins from two sources, both signaling ancient secondary endosymbiotic events involving symbionts from two of the three lineages of Plantae. An ancient "shopping for plastidial eukaryotes" [24] in the heterotrophic ancestor of the heterokonts/chromalveolates could explain the origin of the metabolic versatility that may have subsequently contributed to the ecological success of this group of organisms irrespective of whether photosynthesis was retained or not [77,78]. As in the case of the primary acquisition of chlamydial proteins by the ancestor of the Plantae (see above), it is suggested that in the heterokont/chromalveolate ancestor, genes were also transferred from the old to the new "shopping bag", or to phrase it differently, "you are what you shop".

## Conclusion

We identified 39 proteins of chlamydial origin in photosynthetic eukaryotes. Most likely Chlamydiae invaded the ancestor of the Plantae and intracellular chlamydiae persisted throughout the early history of the Plantae, donating genes either directly or via the cyanobacterial endosymbiont/plastid to their hosts before they eventually vanished. The transferred genes replaced their cyanobacterial/plastid homologs thus shaping early algal/plant evolution. Chlamydial proteins spread through secondary endosymbioses to other photoautotrophic eukaryotes. Heterokonts/chromalveolates seem to have obtained chlamydial proteins from two secondary endosymbiotic events involving symbionts from the rhodoplant and viridiplant lineages.

## Methods

### Data set

We screened for algal proteins of possible chlamydial origin in the following ways: 1) The JGI databases for *Chlamydomonas reinhardtii, Ostreococcus lucimarinus*, *Ostreococcus tauri*, *Phaeodactylum tricornutum *and *Thalassiosira pseudonana *were searched for Blast hits with *Protochlamydia amoebophilia *using the advanced search function and an e-value cut off of exp -20. 2) The proteome of *Cyanidioschyzon merolae *was downloaded from http://merolae.biol.s.u-tokyo.ac.jp/ and blasted (NCBI BLASTP) locally against the proteom of *Protochlamydia amoebophila *using an e-value cut-off of exp-20. Proteins and contigs showing similarity over the entire length were selected for further analysis. To exclude false positive we blasted (BLASTP) each algal protein against the NCB non redundant protein database and the databases for *Chlamydomonas reinhardtii*, *Ostreococcus lucimarinus*, *Ostreococcus tauri*, *Phaeodactylum tricornutum *and *Thalassiosira pseudonana *at JGI and the *Cyanidionschyzon merolae *database at http://merolae.biol.s.u-tokyo.ac.jp/. For proteins showing an association with proteins of Chlamydiae in the distance tree generated by the NCBI BLAST server, we assembled a dataset containing all algal proteins and proteins from *Arabidopsis*, *Oryza*, at least 5 Chlamydiae (incl. *Protochlamydia amoebophilia*), at least 5 cyanobacteria and members of the Proteobacteria and Firmicutes and 5 – 10 bacterial strains obtained as top bacterial BLASTP hits using the protein from *Protochlamydi*a as a query.

### Phylogenetic analysis

Alignment of single-gene data sets were generated using CLUSTAL W and manually refined with SeaView [79]. Non-alignable regions were excluded prior to phylogenetic analyses. The evolutionary model fitting best the protein data was determined with ProtTest 1.2.6 with deactivated "+F" option [80,81]. Maximum likelihood trees were done with phyml 2.4.4 set to the optimal evolutionary model and including 200 bootstrap replicates (in most cases WAG+I+Γ; [82,83]). All trees that were chosen to be depicted, have been subjected in addition to Bayesian inference with MrBayes 3.1.2 [84]. For each data set, two runs with four chains and 3 million generations have been computed. Likelihood parameters were set to 4 gamma categories and proportion of invarable sites. Computation was done across all available amino acid substitution matrices (command "prset aamodel = mixed"). Every 100^th ^generation was sampled. Convergence of the runs was checked according to the output of the "sump" command. The output was also used to determine the burn-in phase.

Two concatenated data sets with a reduced taxon sampling have been assembled from single-gene data. In the first of these data sets, single genes that showed a closer relationship of Bacillariophyta to red algae have been combined, whereas the second data set comprised single genes showing a relationship of Bacillariophyta to Viridiplantae. All concatenated data sets have been subjected to the same analysis procedure as the single genes concerning maximum likelihood analysis (see above). Since in the concatenated data sets some sequences for single taxa were missing, no partitions were defined for Bayesian analyses (see legend of Fig. 3, additional file 5 for details).

## Authors' contributions

BB conceived the study, contributed to its design, performed data analysis, and helped to draft the manuscript. KH–E was responsible for phylogenetic analyses and helped to draft the manuscript. MM conceived the study, contributed to its design, and wrote the manuscript. All authors read and approved the final manuscript.

## Supplementary Material

Additional File 1 Additional Table 1. Proteins from Candidatus *Protochlamydia amoebophila *showing significant similarity to algal proteins. Click here for file

Additional File 2 Additional Table 2. Proteins of putative chlamydial origin in plastid-containing eukaryotes (Extended version of Table 1). Click here for file

Additional File 3 Additional Figure 1. Panels A-F. Click here for file

Additional File 4 Additional Table 3. Comparison of the results of this study with Huang and Gogarten 2007. Click here for file

Additional file 5 Figure legend. Extended legend of Fig. 3. Click here for file
