# Supplementary material for: Chlamydial genes shed light on the evolution of photoautotrophic eukaryotes
Source: BMC Evol Biol. 2008 Jul 15;8:203. doi: 10.1186/1471-2148-8-203 (PMC2490706; doi:10.1186/1471-2148-8-203)
Supplement: Additional File 1 — Additional Table 1. Proteins from Candidatus Protochlamydia amoebophila showing significant similarity to algal proteins. [file 1471-2148-8-203-S1.pdf]

Additional Table 1

Proteins from Candidatus *Protochlamydia amoebophila* showing significant similarity to algal proteins

No	Protein	Accession No.
1.	Phosphate transporter	gi|46445733|ref|YP_007098.1
2.	Putative GTP-binding protein	gi|46447400|ref|YP_008765.1
3.	Putative phosphoglycerate mutase	gi|46399436|emb|CAF22885.1
4.	Hypothetical protein	gi|46446644|ref|YP_008009.1
5.	Putative folylpolyglutamate synthase	gi|46447260|ref|YP_008625.1
6.	Probable ribonucleoside-diphosphate reductase small chain	gi|46446983|ref|YP_008348.1
7.	Transaldolase	gi|46447325|ref|YP_008690.1
8.	Prolyl-tRNA synthetase	gi|46446958|ref|YP_008323.1
9.	Arginyl-tRNA synthetase	gi|46445865|ref|YP_007230.1
10.	Isopentenyl monophosphate kinase (ISPE)	gi|46447223|ref|YP_008588.1
11.	Asparangyl-tRNA synthetase	gi|46445980|ref|YP_007345.1
12.	Probable gcpE protein (ISPG)	gi|46446374|ref|YP_007739.1
13.	Queuine tRNA-ribosyltransferase	gi|46446428|ref|YP_007793.1
14.	Probable naphthoate synthase (menB)	gi|46446698|ref|YP_008063.1
15.	Aspartate aminotransferase	gi|46446319|ref|YP_007684.1
16.	Peptide chain release factor 1	gi|46446286|ref|YP_007651.1
17.	Probable ribonucleoside-diphosphate reductase large chain	gi|46446982|ref|YP_008347.1
18.	Hypothetical protein pc0695	gi|46446329|ref|YP_007694.1
19.	Probable triose-phosphate isomerase	gi|46446435|ref|YP_007800.1
20.	Hypothetical protein pc0225	gi|46445859|ref|YP_007224.1
21.	Putative 7-dehydrocholesterol reductase	gi|46446854|ref|YP_008219.1
22.	ATP/ADP translocase	
23.	Transketolase	gi|46447148|ref|YP_008513.1
24.	Enoyl-(acyl carrier protein) reductase (FABI)	gi|46446786|ref|YP_008151.1
25.	Putative 23S rRNA (Uracil-5-)-methyltransferase	gi|46447632|ref|YP_008997.1
26.	Putative 4-diphosphocytidyl-2C-methyl-D-erythritol synthase (ISPD)	gi|46445961|ref|YP_007326.1
27.	DNA mismatch repair protein (MUTS)	gi|46446855|ref|YP_008220.1
28.	Hypothetical protein pc1982	gi|46447616|ref|YP_008981.1
29.	Hpothetical protein pc0264	gi|46445898|ref|YP_007263.1
30.	Hypothetical protein pc1328	gi|46446962|ref|YP_008327.1
31.	Putative 1,4-dihydroxy-2-naphthoate octaprenyltransferase (menA)	gi|46446697|ref|YP_008062.1
32.	Putative lipoate-protein ligase	gi|46447472|ref|YP_008837.1
33.	Ribonuclease HII	gi|46446292|ref|YP_007657.1
34.	tRNA delta(2)-isopentenylpyrophosphate transferase	gi|46446877|ref|YP_008242.1
35.	Putative Na^+/H^+ antiporter	gi|46445728|ref|YP_007093.1
36.	Putative eucaryotic NAD-specific glutamate dehydrogenase	gi|46447130|ref|YP_008495.1
37.	Probable superoxide dismutase (Mn)	gi|46445904|ref|YP_007269.1

38.	Putative glycerol-3-phosphate acyltransferase	gi|46446952|ref|YP_008317.1
39.	Hypothetical protein pc1271	gi|46446905|ref|YP_008270.1
40.	Probable 3,4-dihydroxy-2-butanone 4-phosphate synthase/GTP cyclohydrolase II	gi|46446524|ref|YP_007889.1
41.	Probable 23S RNA-specific pseudouridine synthase D	gi|46445989|ref|YP_007354.1
42.	Probable SufS L-cysteine desulfurase/L-selenocysteine lyase	gi|46445826|ref|YP_007191.1
43.	putative glutamate-ammonia ligase (=glutamine synthetase) type III	gi|46446874|ref|YP_008239.1
44.	Putative Thermostable carboxypeptidase 1	gi|46446264|ref|YP_007629.1
45.	Probable glutamyl-tRNA(Gln) amidotransferase chain A	gi|46446304|ref|YP_007669.1
46.	Probable polyribonucleotide nucleotidyltransferase	gi|46446277|ref|YP_007642.1
47.	Probable signal recognition particle	gi|46446933|ref|YP_008298.1
48.	Putative inositol-1(or 4)-monophosphatase	gi|46447210|ref|YP_008575.1
49.	Hypothetical protein pc0339	gi|46445973|ref|YP_007338.1
50.	Hypothetical protein pc0673	gi|46446307|ref|YP_007672.1
51.	Hypothetical protein pc0264	gi|46445898|ref|YP_007263.1
52.	Putative 3-dehydroquinate synthase	gi|46445707|ref|YP_007072.1
53.	Conserved hypothetical protein	gi|46400983|emb|CAF24432.1
54.	Putative Gut Q protein	gi|46447416|ref|YP_008781.1
55.	Putative cadmium-transporting ATPase	gi|46445921|ref|YP_007286.1
56.	Leucyl aminopeptidase	gi|46446737|ref|YP_008102.1
57.	Glycogen synthase	gi|46447230|ref|YP_008595.1
58.	1-deoxy-D-xylulose 5-phosphate reductoisomerase	gi|46445894|ref|YP_007259.1
59.	Leucyl-tRNA synthetase	gi|46446681|ref|YP_008046.1
60.	Aspartyl-tRNA synthetase	gi|46446018|ref|YP_007383.1
61.	Malate dehydrogenase	gi|46447406|ref|YP_008771.1
62.	Probable S-adenosyl-methyltransferase	gi|46445945|ref|YP_007310.1
63.	Putative tRNA pseudouridylate synthase I	gi|46445962|ref|YP_007327.1
64.	Hypothetical protein pc1271	gi|46446905|ref|YP_008270.1
65.	3-oxoacyl-(acyl carrier protein) synthase (FABB)	gi|46446872|ref|YP_008237.1
66.	Putative ribosome recycling factor	gi|46447510|ref|YP_008875.1
67.	Putative endopeptidase (ATP-dependent serine protease) La	gi|46446096|ref|YP_007461.1
68.	Putative tyrosine/tryptophan transport protein	gi|46445802|ref|YP_007167.1
69.	Putative 4-alpha-glucanotransferase	gi|46446379|ref|YP_007744.1
70.	Aspartate aminotransferase	gi|46446319|ref|YP_007684.1
71.	Putative photolyase	gi|46446306|ref|YP_007671.1
72.	Hypothetical protein pc0324	gi|46445958|ref|YP_007323.1
73.	Hypothetical protein pc0378	gi|46446012|ref|YP_007377.1
74.	Hypothetical protein pc1982	gi|46447616|ref|YP_008981.1
75.	Hypothetical protein pc0141	gi|46445775|ref|YP_007140.1
76.	Chorismate synthase	gi|46446518|ref|YP_007883.1
77.	Probable heat shock protein dnaJ	gi|46446102|ref|YP_007467.1
78.	Hypothetical protein pc1987	gi|46447621|ref|YP_008986.1

79.	Probable tyrosine-tRNA ligase	gi|46446803|ref|YP_008168.1
80.	Probable thioredoxin-disulfide reductase 2	gi|46447347|ref|YP_008712.1
81.	Probable isoamylase	gi|46446740|ref|YP_008105.1
82.	Glycogen synthase	gi|46447230|ref|YP_008595.1
83.	Hypothetical protein pc0648	gi|46446282|ref|YP_007647.1
84.	Probable 3-deoxy-manno-octulosonate cytidylyltransferase (CMP-KDO synthetase)	gi|46400100|emb|CAF23549.1
85.	Diphosphate--fructose-6-phosphate 1-phosphotransferase	gi|46446514|ref|YP_007879.1
86.	Putative 23S rRNA (Uracil-5-)-methyltransferase	gi|46447632|ref|YP_008997.1
87.	Cysteinyl-tRNA synthetase	gi|46446869|ref|YP_008234.1
88.	Probable S-adenosyl-methyltransferase	gi|46445945|ref|YP_007310.1
89.	Putative oligoendopeptidase F	gi|46446812|ref|YP_008177.1
