# Supplementary material for: Chlamydial genes shed light on the evolution of photoautotrophic eukaryotes
Source: BMC Evol Biol. 2008 Jul 15;8:203. doi: 10.1186/1471-2148-8-203 (PMC2490706; doi:10.1186/1471-2148-8-203)
Supplement: Additional File 2 — Additional Table 2. Proteins of putative chlamydial origin in plastid-containing eukaryotes (Extended version of Table 1). [file 1471-2148-8-203-S2.pdf]

Table 1 - Proteins of putative chlamydial origin in plastid-containing eukaryotes.

39 proteins were identified by ML analyses. The proteins are grouped according to the observed tree topologies in the maximum likelihood analyses. The bootstrap support (ML) for the indicated clades is given. For 20 genes (in bold letters) this is to our knowledge the first detailed phylogenetic analysis.

	Gene[1)]	Figure	Absent in	Topology[2)]	Comments
**(Chlamydiae, Plantae)**					
1	**Asparaginyl-tRNA synthetase**<br>gi|46445980		C	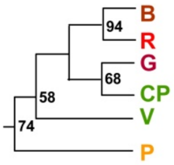	
2	Aspartate aminotransferase<br>gi|46446319			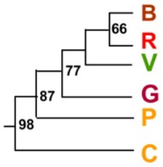	(**CB** **F)** 100% ML
3	ATP/ADP translocase		**CB**	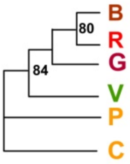	Only in intracellular parasites and plastid-containing eukaryotes
4	**tRNA delta(2)-isopentenylpyro-phosphate transferase**<br>gi|46446877	**2 B**		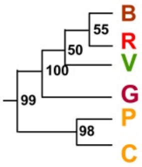	
5	**Diphosphate--fructose-6-phosphate 1-phosphotransferase**<br>gi|46446514		**CB**	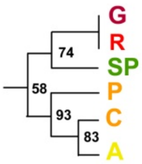	

[1)] The accession number and annotation for the gene from *Protochlamydia* is given.
[2)]Topology: A Apicomplexa, B Bacillariophyta, C Chlamydiae except Protochlamydia, CB Cyanobacteria, CP Chlorophyta, F Firmicutes, G Glaucoplantae, P Protochlamydia, R Rhodoplantae, SP Streptophyta, V Viridiplantae.

Table 1 continued

	Gene[1)]	Figure	Absent in	Topology[2)]	Comments
**(Chlamydiae, Viridiplantae, Rhodoplantae)**					
6	Isopentenyl monophosphate kinase (ISPE) gi|46447223			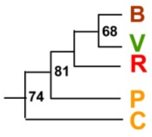	
7	**Queuine tRNA-ribosyltransferase** gi|46446428			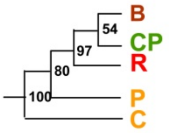	*Dictyostelium* sister to **[P]**
8	**Putative 7-dehydrocholesterol reductase** gi|46446854		**CB, C**	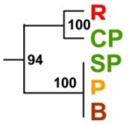	Only eukaryotes except *Protochlamydia* and *Coxiella*, *Coxiella* sister to *Protochlamydia* (87% ML)
9	Putative 23S rRNA (Uracil-5-)-methyltransferase gi|46447632		**C**	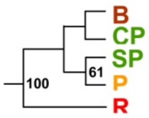	
10	Putative 4-diphosphocytidyl-2C-methyl-D-erythritol synthase (ISPD) gi|46445961	**2 C**		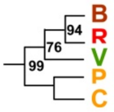	**(CB F)** 73% ML
11	**Hypothetical protein pc1328** gi|46446962			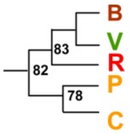	
12	Putative glycerol-3-phosphate acyltransferase gi|46446952	**2 A**	**CB**	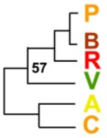	Only present in Chlamydiae and plastid containing eukaryotes
13	Probable polyribonucleotide nucleotidyltransferase gi|46446277			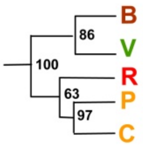	**(CB F)** 100% ML
14	**Probable S-adenosyl-methyltransferase** gi|46445945			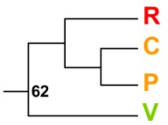	
15	**Putative tRNA pseudouridylate synthase I** gi|46445962			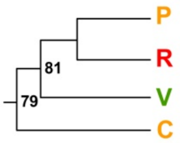	

16	3-oxoacyl-(acyl carrier protein) synthase (FABB) gi|46446872		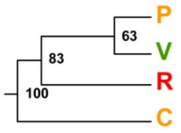	Bacillariophyceae in eubacterial clade
17	**Putative endopeptidase (ATP-dependent serine protease) La** gi|46446096	**CB**	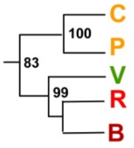	
18	Probable tyrosine-tRNA ligase gi|46446803		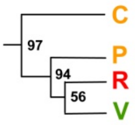	Bacillariophyceae in bacterial clade
19	Probable isoamylase gi|46446740		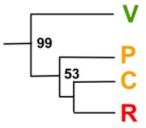	
20	**Probable S-adenosyl-methyltransferase** gi|46445945	C	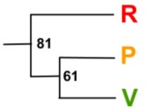	Bacillariophyceae in eukaryotic clade
21	Putative oligoendopeptidase F gi|46446812	**CB**	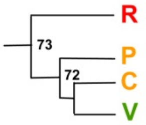	

[1] The accession number and annotation for the gene from *Protochlamydia* is given.
[2]Topology: A Apicomplexa, B Bacillariophyceae, C Chlamydiae except Protochlamydia, CB Cyanobacteria, CP Chlorophyta, F Firmicutes, G Glaucoplantae, P Protochlamydia, R Rhodoplantae, SP Streptophyta, V Viridiplantae.

Table 1 continued

	Gene[1)]	Figure	Absent in	Topology[2)]	Comments
**(Chlamydiae, Viridiplantae)**					
**22**	**Phosphate transporter gi|46445733**			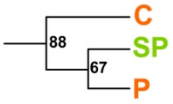	
23	Phosphoglycerate mutase gi|46399436		**R**	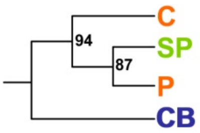	
24	Probable gcpE protein (ISPG) gi|46446374			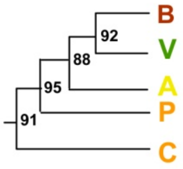	**(CB G R)** 100% ML
25	Enoyl-(acyl carrier protein) reductase (FABI) gi|46446786			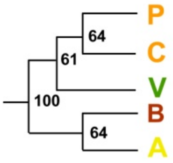	**(CB R)**
26	**DNA mismatch repair protein (MUTS)** gi|46446855			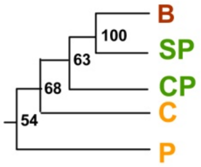	**(CB R)** 95% ML
27	**Putative lipoate-protein ligase** gi|46447472		**R, CB**	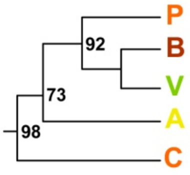	*Dictyostelium* sister to **[B V]**
28	Gut Q protein gi|46447416		**R**	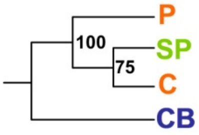	
29	Malate dehydrogenase gi|46447406		**R**	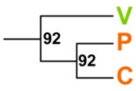	$NADP^+$-dependent plastidial homologue of the Viridiplantae

30	**Putative ribosome recycling factor** gi|46447510	**2 D**	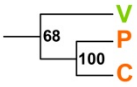	(**CB** **R** **G**) 88% ML
31	**Putative tyrosine/tryptophan transport protein** gi|46445802	**CB**, **R**	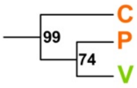	
32	**Hypothetical protein pc0324** gi|46445958	**C**, **R**, **CB**	Not applicable	Only present in Viridiplantae and Protochlamydia
33	**Hypothetical protein pc0378** gi|46446012|	**R**	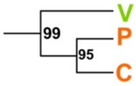	
34	Probable 3-deoxy-manno-octulosonate cytidylyltransferase (CMP-KDO synthetase) gi|46400100	B	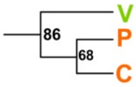	

[1)] The accession number and annotation for the gene from *Protochlamydia* is given.
[2)]Topology: A Apicomplexa, B Bacillariophyceae, C Chlamydiae except Protochlamydia, CB Cyanobacteria, CP Chlorophyta, F Firmicutes, G Glaucoplantae, P Protochlamydia, R Rhodoplantae, SP Streptophyta, V Viridiplantae.

Table I continued

	Gene[1)]	Figure	Absent in	Topology[2)]	Comments
**(Chlamydiae, Rhodoplantae)**					
35	**Probable 23S RNA-specific pseudouridine synthase D** gi|46445989	**2 E**		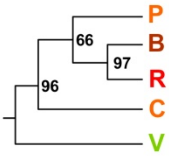	
36	**Hypothetical protein pc0339** gi|46445973		C	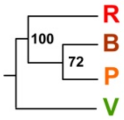	*Geobacter* sister to [V]
37	**Cysteinyl-tRNA synthetase** gi|46446869			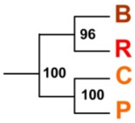	*Leptosira* sister to **[C P]**
**(Chlamydiae, Bacillariophyceae)**					
38	**Putative folylpolyglutamate synthase** gi|46447260	**2 F**	C	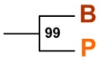	
39	Transketolase gi|46447148			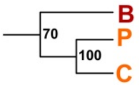	*Dictyostelium* sister to [B], **[V G CB]**

1) The accession number and annotation for the gene from *Protochlamydia* is given.
2)Topology: A Apicomplexa, B Bacillariophyceae, C Chlamydiae except Protochlamydia, CB Cyanobacteria, CP Chlorophyta, F Firmicutes, G Glaucoplantae, P Protochlamydia, R Rhodoplantae, SP Streptophyta, V Viridiplantae.
