# Supplementary material for: Chlamydial genes shed light on the evolution of photoautotrophic eukaryotes
Source: BMC Evol Biol. 2008 Jul 15;8:203. doi: 10.1186/1471-2148-8-203 (PMC2490706; doi:10.1186/1471-2148-8-203)
Supplement: Additional File 3 — Additional Figure 1. Panels A-F. [file 1471-2148-8-203-S3.pdf]

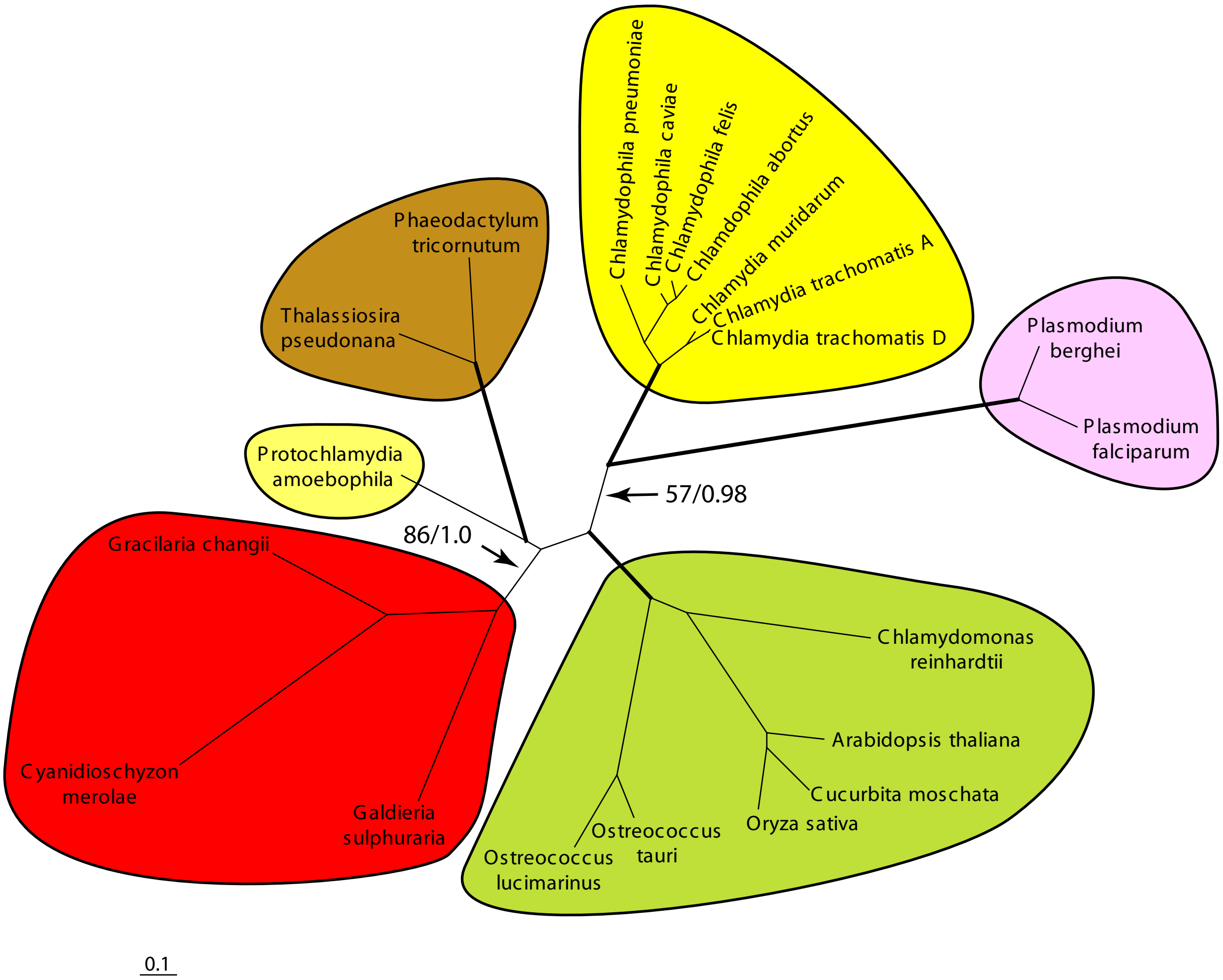

Additional Figure 1A. Glycerol-3-phosphate acyltransferase (EC 2.3.1.15)

21 taxa, 281 amino acid positions. Color code: yellow, Chlamydiae; red, Rhodoplantae; green, Viridiplantae; brown, Bacillariophyta; light purple, Apicomplexa. Evolutionary model: WAG+I+Γ. Support values: maximum likelihood bootstrap/posterior probabilities; branches in bold: maximum likelihood bootstrap > 95% and posterior probability = 1.0.

Scale bar = substitutions per site

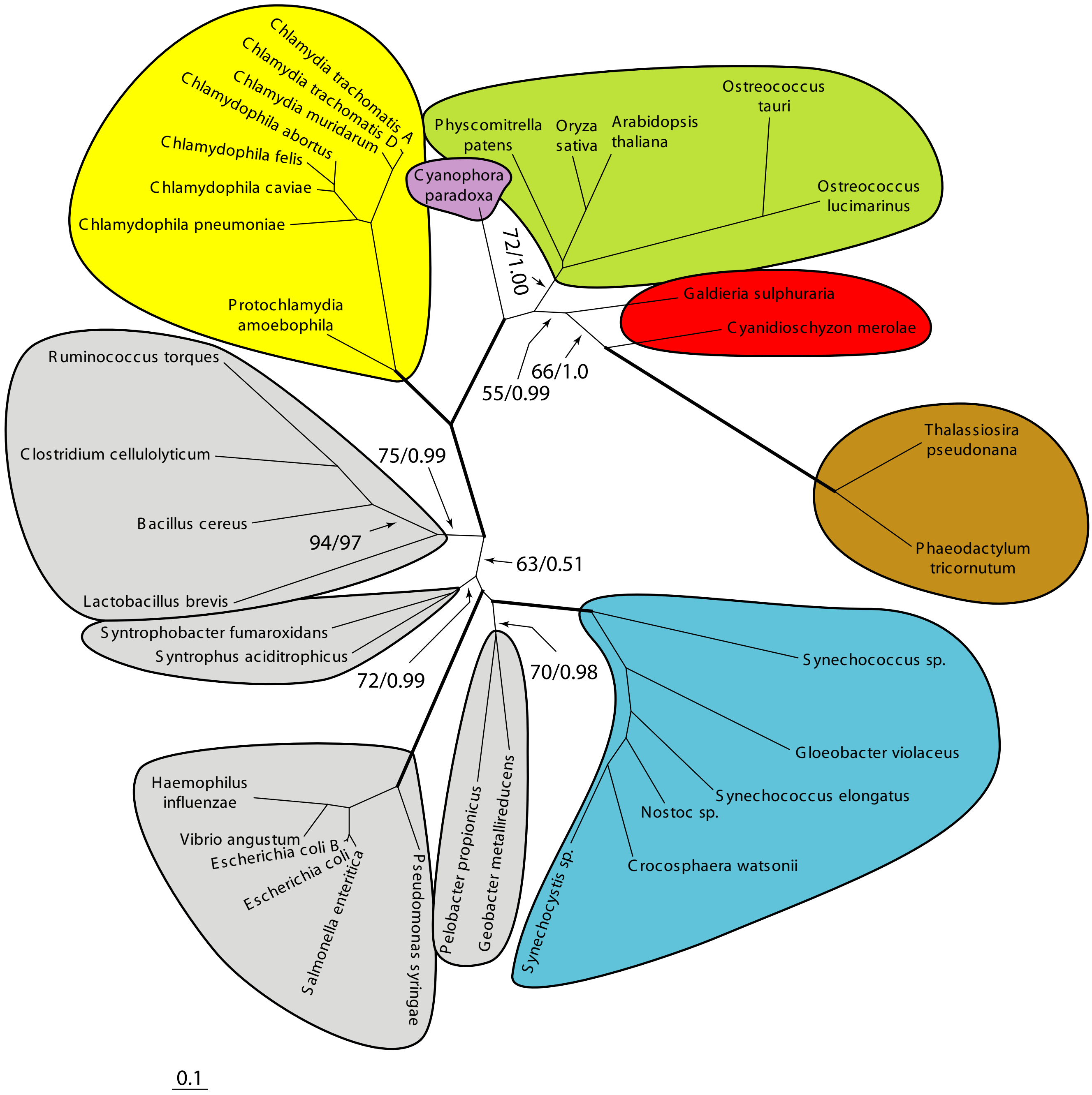

Additional Figure 1B. tRNA delta(2)-isopentenylpyrophosphate transferase (EC 2.5.2.8) 38 taxa, 240 positions. Color code: yellow, Chlamydiae; red, Rhodoplantae; green, Viridiplantae; purple, Glaucoplantae; brown, Bacillariophyta; blue, Cyanobacteria; gray, other bacteria. Evolutionary model: WAG+I+Γ. Support values: maximum likelihood bootstrap/posterior probabilities; branches in bold: maximum likelihood bootstrap > 95% and posterior probability = 1.0.
Scale bar = substitutions per site

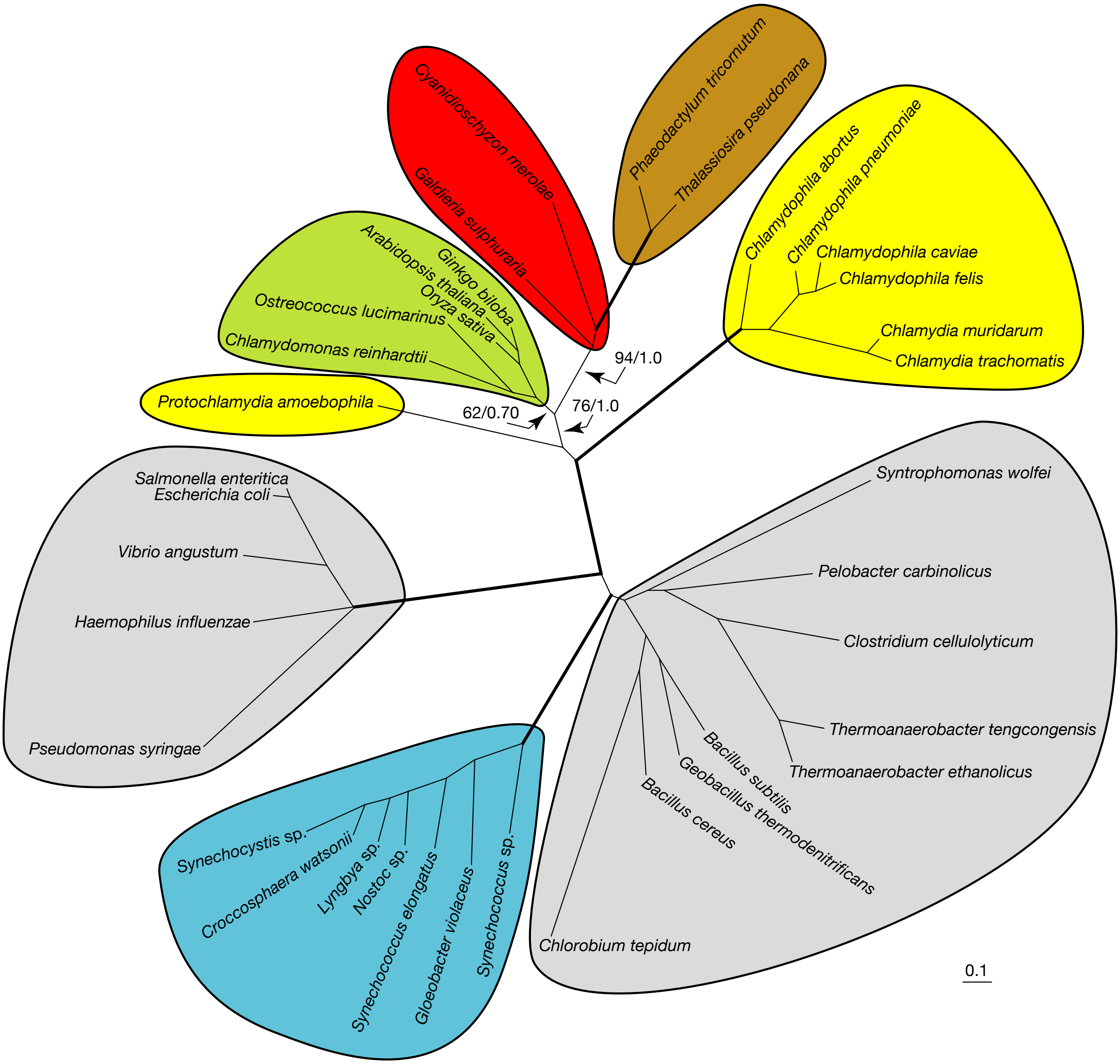

Additional Figure 1C. 2-C-methyl-D-erythritol 4-phosphate cytidyltransferase (*isp*D; EC 2.7.7.60). 38 taxa, 198 amino acid positions. Color code: yellow, Chlamydiae; red, Rhodoplantae; green, Viridiplantae; brown, Bacillariophyta. Evolutionary model: RtREV+I+Γ. Support values: maximum likelihood bootstrap/posterior probabilities; branches in bold: maximum likelihood bootstrap > 95% and posterior probability = 1.0. Scale bar = substitutions per site

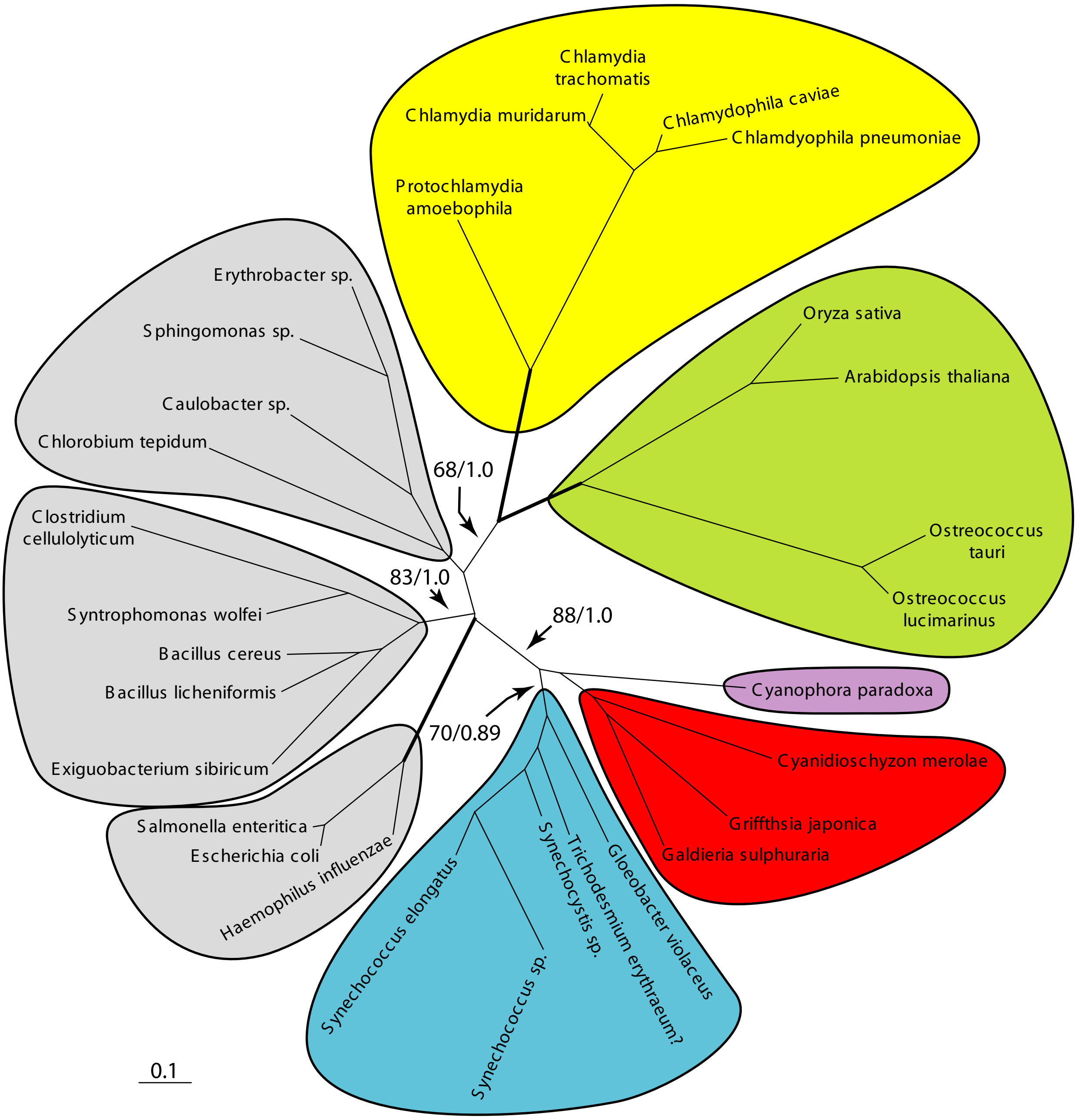

Additional Figure 1D. Putative ribosome release/recycling factor (COG0233).
30 taxa, 171 positions. Color code: yellow, Chlamydiae; green, Viridiplantae; red, Rhodoplantae; brown, Bacillariophyta; purple, Glaucoplantae; blue, Cyanobacteria; gray, other bacteria. Evolutionary model: WAG+I+Γ. Support values: maximum likelihood bootstrap/posterior probability; branches in bold: ML bootstrap > 95% and posterior probability of 1.0.
Scale bars = substitutions per site.

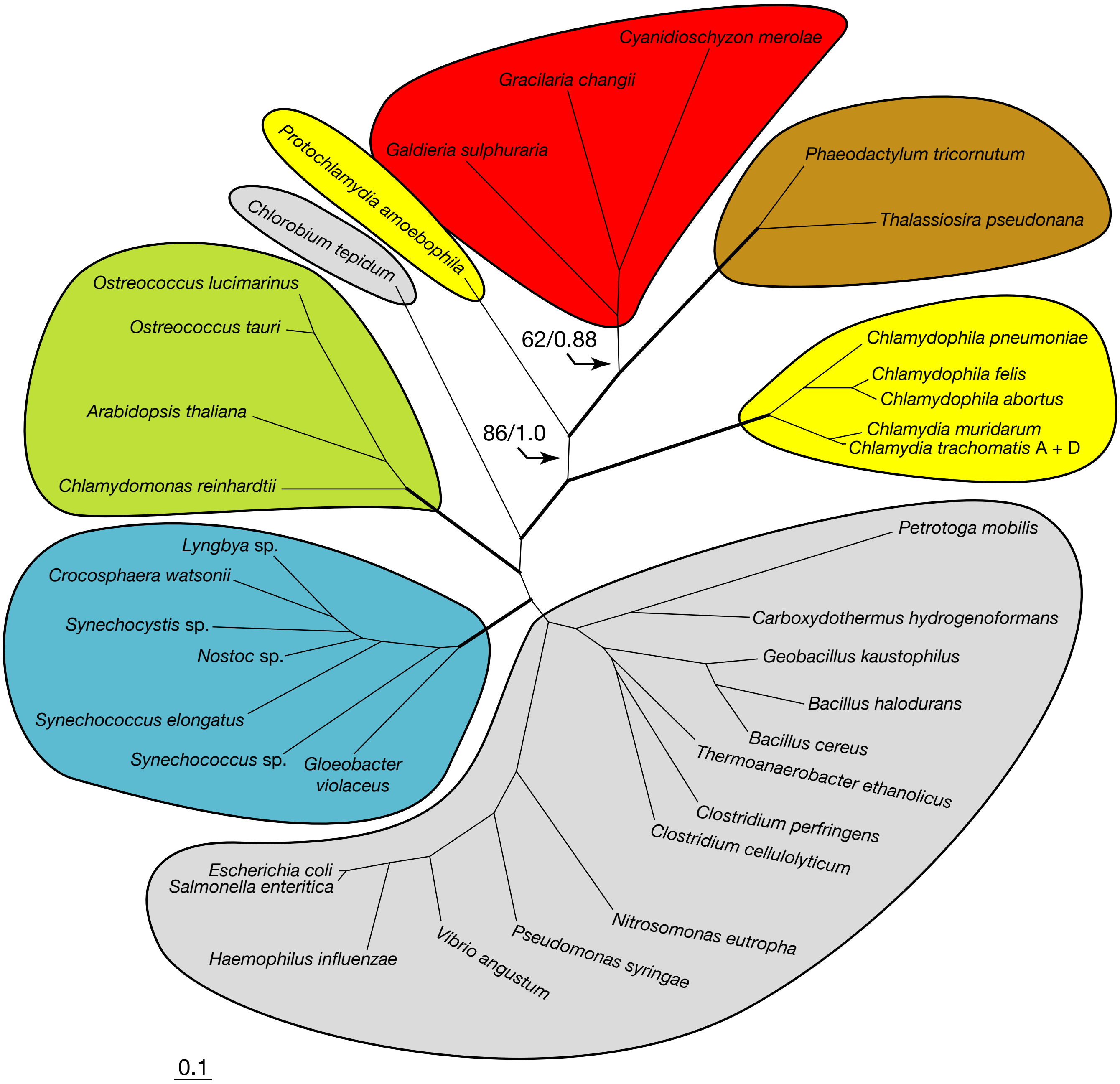

Additional Figure 1E. Ribosomal large subunit pseudouridine synthase (EC 4.2.1.70). 38 taxa, 262 positions. Color code: yellow, Chlamydiae; red, Rhodoplantae; green, Viridiplantae; brown, Bacillariophyta; blue, Cyanobacteria; gray, other bacteria. Evolutionary model: RtREV+I+Γ. Support values: maximum likelihood bootstrap/posterior probabilities; branches in bold: maximum likelihood bootstrap > 95% and posterior probability = 1.0.

Scale bar = substitutions per site

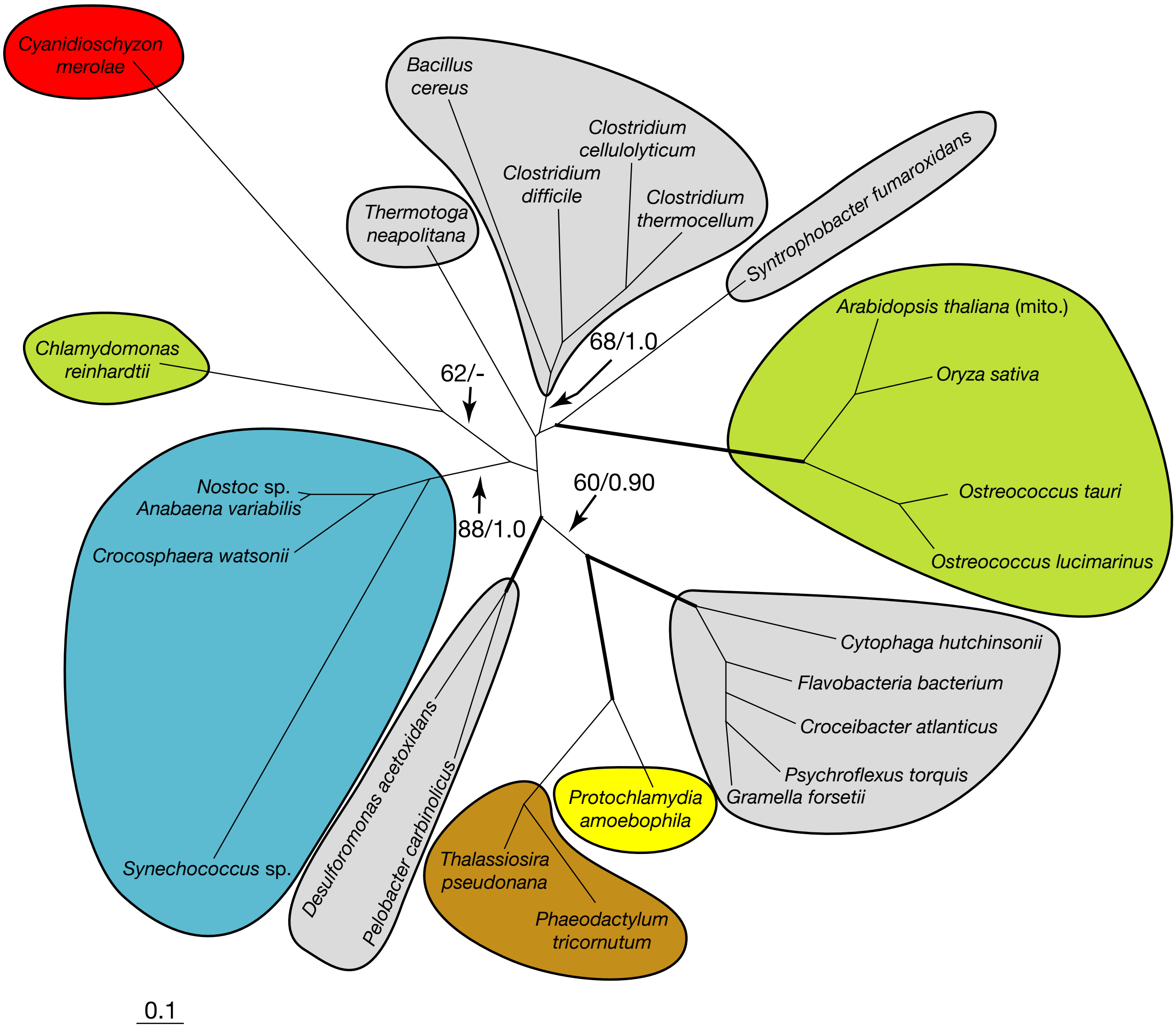

Additional Figure 1F. Folylpolyglutamate synthase (EC 6.3.2.17).
26 taxa, 208 positions. Color code: yellow, Chlamydiae; red, Rhodoplantae; green, Viridiplantae; brown, Bacillariophyta; blue, Cyanobacteria; gray, other bacteria.
Evolutionary model: WAG+I+Γ. Support values: maximum likelihood bootstrap/posterior probabilities; branches in bold: maximum likelihood bootstrap > 95% and posterior probability = 1.0.
Scale bar = substitutions per site
