# Supplementary material for: Chlamydial genes shed light on the evolution of photoautotrophic eukaryotes
Source: BMC Evol Biol. 2008 Jul 15;8:203. doi: 10.1186/1471-2148-8-203 (PMC2490706; doi:10.1186/1471-2148-8-203)
Supplement: Additional File 4 — Additional Table 3. Comparison of the results of this study with Huang and Gogarten 2007. [file 1471-2148-8-203-S4.pdf]

**Additional Table 3: Comparison of the results of this study with Huang and Gogarten 2007** [1]

| | This study | Table 1 [1] |
|---|---|---|
| No of chlamydial proteins in red algae[1)] | 24 | 16 |
| Chlamydial protein[1)]: | | |
| Sodium hydrogen antiporter | - | + |
| Cu ATPase | - | + |
| Different interpretation: | | |
| Oligoendopeptidase F[2)] | R and V | R |
| *fab*I[3)] | V | R and V |
| *gcp*E[3)] | V | R and V |

[1)] There are two major differences between this work and that of Huang and Gogarten [1]: (1) The taxon sampling is broader, especially including more algal sequences and (2) whereas Huang and Gogarten 2007 [1] used a single evolutionary model for all analyses, in this study a model test was performed prior to phylogenetic analyses and the best model chosen for the analyses. We believe that both factors contributed to the observed differences.

[2)] The chlamydial-type oligoendopeptidase F reported by [1] to be restricted to Rhodoplantae (R) and Chlamydiae is also present in the Viridiplantae (V). Clear homologues for this protein were found in *Ostreococcus tauri*, *Ostreococcus lucimarinus* and *Mesostigma viride*, which formed a clade together with the protein from Rhodoplantae and are sister to Chlamydiae.

[3)] For both proteins (*gcp*E = *isp*G and *fab*I) the present analyses indicated only a sister relationship between the protein from the Viridiplantae and Chlamydiae, excluding Rhodoplantae. In the protein trees in both studies only the proteins from the Bacillariophyta form a clade together with the Viridiplantae. The proteins from the Rhodoplantae form a clade with cyanobacterial proteins (see Results).

References


1. Huang, J., and Gogarten, P. (2007). Did an ancient chlamydial endosymbiosis facilitate the establishment of primary plastids? Genome Biology *8*, R99.
