# Supplementary material for: Chlamydial genes shed light on the evolution of photoautotrophic eukaryotes
Source: BMC Evol Biol. 2008 Jul 15;8:203. doi: 10.1186/1471-2148-8-203 (PMC2490706; doi:10.1186/1471-2148-8-203)
Supplement: Additional file 5 — Figure legend. Extended legend of Fig. 3. [file 1471-2148-8-203-S5.pdf]

Figure 3 - Phylogenetic analyses of chlamydial proteins in the Bacillariophyta support the occurrence of two independent HGT/EGT events.

Unrooted maximum likelihood trees of concatenated data sets. Support values: maximum likelihood bootstrap/posterior probability. Scale bars = substitutions per site.

(**A**) Concatenated data set of seven proteins showing relationship of diatoms to rhodoplants (12 taxa; 2675 amino acid positions). The data set consisted of asparaginyl-tRNA synthetase (*asn*S; EC 6.1.1.22; no *Chlamydophila caviae* and *Chlamydia muridarum* sequences, No 1 in Table 1), aspartate aminotransaminase (*aat*; EC 2.6.1.1, No 2 in Table 1), ATP/ADP translocase 1 (*tlc*1, *ntt*1; COG3202; only the orthologues of the nucleotide transporter family have been chosen, No 3 in Table 1), tRNA delta(2)-isopentenylpyrophosphate transferase (*mia*A; EC 2.5.1.8; no *Chlamydomonas reinhardtii* sequence, No 4 in Table 1), putative 4-diphosphocytidyl-2C-methyl-D-erythritol synthase (*isp*D; EC 2.7.7.60; no *Ostreococcus tauri* sequence No 10 in Table 1), putative glycerol-3-phosphate O-acyltransferase (*gpa*T; EC 2.3.1.15, No 12 in Table 1) and putative endopeptidase La (ATP-dependent serine protease) La (*lon*; EC 3.4.21.53; no *Ostreococcus tauri* sequence, No 17 in Table 1).

(**B**) Concatenated data set of five proteins showing a relationship of viridiplant and diatom genes (same 12 taxa as in Fig. 3A; 1736 amino acid positions). The data set contained Isopentenyl monophosphate kinase (*isp*E; EC 2.7.1.148; no *Galdieria sulphuraria* sequence, No 6 in Table 1), queuine tRNA-ribosyltransferase (*tgt*; EC 2.4.2.29; no *Oryza sativa* and *Arabidopsis thaliana* sequences, No 7 in Table 1), putative 23S rRNA (Uracil-5-)-methyltransferase (*trm*A; EC 2.1.1.53; no *Chlamydophila caviae*, *Chlamydia muridarum* and *Chlamydomonas reinhardtii* sequences, No 9 in Table 1), hypothetical protein pc1328 (COG0217, No 11 in Table 1) and probable polyribonucleotide nucleotidyltransferase (*pnp*; EC 2.7.7.8, No 13 in Table 1).
